# Putative climate adaptation in American pikas (*Ochotona princeps*) is associated with copy number variation across environmental gradients

**DOI:** 10.1038/s41598-024-59157-6

**Published:** 2024-04-13

**Authors:** Bryson M. F. Sjodin, Danielle A. Schmidt, Kurt E. Galbreath, Michael A. Russello

**Affiliations:** 1 Department of Biology, The University of British Columbia, 3247 University Way, Kelowna, BC V1V 1V7 Canada; 2 Department of Biology, Northern Michigan University, 1401 Presque Isle Ave, Marquette, MI 49855 USA

**Keywords:** Copy number variant, Local adaptation, Climate, RADseq, Latitudinal and elevational gradients, Structural variation, Conservation genomics

## Abstract

Improved understanding of the genetic basis of adaptation to climate change is necessary for maintaining global biodiversity moving forward. Studies to date have largely focused on sequence variation, yet there is growing evidence that suggests that changes in genome structure may be an even more significant source of adaptive potential. The American pika (*Ochotona princeps*) is an alpine specialist that shows some evidence of adaptation to climate along elevational gradients, but previous work has been limited to single nucleotide polymorphism based analyses within a fraction of the species range. Here, we investigated the role of copy number variation underlying patterns of local adaptation in the American pika using genome-wide data previously collected across the entire species range. We identified 37–193 putative copy number variants (CNVs) associated with environmental variation (temperature, precipitation, solar radiation) within each of the six major American pika lineages, with patterns of divergence largely following elevational and latitudinal gradients. Genes associated (*n* = 158) with independent annotations across lineages, variables, and/or CNVs had functions related to mitochondrial structure/function, immune response, hypoxia, olfaction, and DNA repair. Some of these genes have been previously linked to putative high elevation and/or climate adaptation in other species, suggesting they may serve as important targets in future studies.

## Introduction

The complex interplay between environmental factors and local adaptation plays an essential role in the generation and maintenance of biodiversity^1–4^. Typically, genetic studies on adaptation have focused on DNA sequences (single nucleotide polymorphisms; SNPs), given that point mutations were thought to be the predominant source of selectable variation^5,6^; however, genomes can also vary in their physical structure across species, populations, and even individuals^7–12^. These physical variations, collectively known as structural variants, include insertions or deletions of single or large numbers of nucleotides, duplications of genes or entire regions of the genome, inversions or changes in polarity of chromosomes, and translocations both within and among chromosomes. Structural variants can be significant factors in shaping species divergence and local adaptation^10,12–16^. Early comparative genomics research determined that chromosomal inversions were linked to speciation in *Drosophila*^8^. More recent work is focused on the eco-evolutionary impact of structural variation within natural populations^12,16^. For instance, Arostegui et al*.*^17^ found that a chromosomal inversion is likely responsible for ecotype differentiation in rainbow trout. Likewise, Cayuela et al*.*^13^ found significant genotype-environment associations (GEA) among sympatric capelin lineages in the North Atlantic Ocean linked to chromosomal rearrangements and hypothesized that they were associated with adaptation to environmental conditions at spawning sites. These examples and others demonstrate how investigations of structural variants can provide important and novel insights into local adaptation.

Copy number variants (CNVs) span different classes of structural variants (e.g., insertions/deletion, duplications, transposable elements) that vary in the number of copies among individuals^18^. Previous studies have revealed that signal at CNVs can differ relative to SNPs, providing important insights into population differentiation and evolutionary history, including the genetic basis of adaptation (reviewed in Mérot et al.^10^). Importantly, recent studies have effectively detected and analyzed CNVs from reduced representation genome sequencing (e.g., restriction-site associated DNA sequencing; RADseq^19^), providing a cost-effective approach for investigating the role of structural variation in a range of non-model organisms, including American lobster^20^, capelin^21^, and Columbia spotted frog^22^. Moreover, these analytical approaches can now be retrospectively applied to the wealth of publicly available RADseq data to investigate the potential role of CNVs underlying patterns of ecological and evolutionary relevance^23^.

The American pika (*Ochotona princeps*) is a small lagomorph distributed across a large latitudinal gradient in western North America, from New Mexico (USA) to central British Columbia (Canada)^24,25^. Pikas are alpine specialists typically found at elevations > 2000 m, although their full distribution spans an elevational gradient from 0 to 4000 m^25–27^. These environmental gradients serve as an excellent system for investigating local adaptation, as variations in genomic architecture can be directly correlated to differences in environmental variables including temperature, precipitation, and solar radiation^1,28^. Furthermore, American pikas can also be separated into six geographically isolated evolutionary lineages^29^, allowing for investigations of potentially independent and parallel histories of adaptation within the same system. While previous studies have found genetic evidence for local adaptation in the American pika on regional scales^30–32^ and at the whole genome level^33,34^, range-wide adaptation has not been explored. Moreover, these previous studies focused solely on sequence variation; investigation of structural variation may provide a novel, yet complementary, perspective on local adaptation in this species.

To complement past and on-going studies of sequence variation, we investigated the role of CNVs underlying patterns of local adaptation in the American pika using RADseq data previously collected for 36 sites across the species range^29^. We used a combination of partial redundancy analysis and linear mixed-effect modelling to identify loci putatively associated with temperature, precipitation, and solar radiation within and across all six major American pika lineages. We subsequently examined the spatial distribution of putatively adaptive variation and population differentiation across range-wide latitudinal and elevational gradients. Finally, we annotated putatively adaptive variants and identified target genes with potential functional impacts on local adaptation.

## Methods

### Data and study area

**Figure 1:**
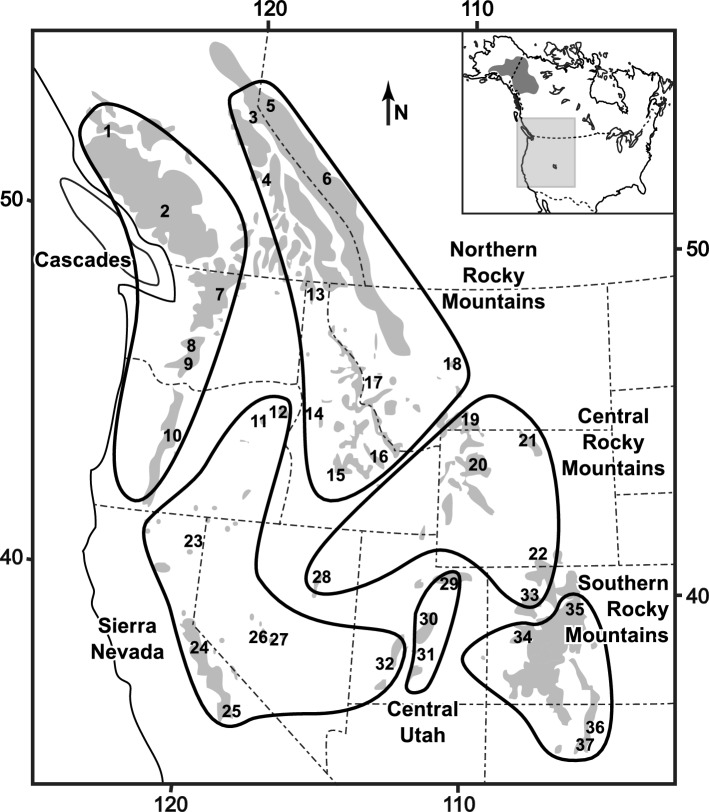
Sampling sites and lineage delineations (thick black lines) for the American pika (*Ochotona princeps*). Shaded regions indicate the approximate American pika distribution as modified from Galbreath et al*.*^35^.

We used previously generated RADseq data^29^ collected from 348 individuals sampled from 36 localities across the entire American pika distribution spanning all six major lineages: (NRM) Northern Rocky Mountains; (CRM) Central Rocky Mountains; (SRM) Southern Rocky Mountains; (CSC) Cascades; (SN) Sierra Nevada; and (CU) Central Utah (Fig. 1; Table S1); a subset (n = 173) of these samples were used in Galbreath et al*.*^35^. We removed site 18 from the NRM lineage as this site displayed significant admixture between the NRM and CRM lineages^29^.

### Initial SNP calling and filtering

Raw sequencing data from all 11 RADseq libraries were de-multiplexed using the *process_radtags* module of Stacks *v*2.57^36^; during this stage, reads were trimmed to 94 bp to remove low-quality bases at the end of the read. De-multiplexed sequences were then aligned to the American pika reference genome assembly (OchPri4.0; GenBank accession ID: GCA_014633375.1^33^) using BWA-mem *v*2.2.1^37^ under default parameters. The aligned sequence data was then processed using the *gstacks* module of Stacks *v*2.57^36^ to generate a catalog of RADtags for downstream analyses.

Due to low range-wide heterozygosity^38,39^ that interfered with downstream copy number variant (CNV) detection (see Discussion for further explanation), we called SNPs independently within each lineage from the aligned sequence data using Stacks v2.57^36^ retaining polymorphic, autosomal SNPs present in ≥ 70% of all individuals within that lineage. This design also allowed us to assess patterns of parallel evolution by considering each lineage as a separate pseudoreplicate. We further filtered loci to only retain genotypes with a genotyping quality ≥ 20 using VCFtools v0.1.15^40^ and only retained loci present in ≥ 2 individuals within each sample site. Following Dorant et al*.*^20^, we performed a final filtering step to retain loci which genotyped in ≥ 70% of individuals within a sample site (allowing for 10% of populations to fail this threshold; maximum of 2 populations per lineage) and had a minimum minor allele sample (i.e., the minimum number of samples possessing the minor/rare allele) of 5% of individuals within a lineage (rounded up to the nearest individual) across all individuals within a lineage using the *05_filter_vcf_fast.py* script downloaded from https://github.com/enormandeau/stacks_workflow (see Table S1 for a summary of filtering values). Also during this step, individual genotypes at a locus with a total read depth < 4 (i.e., minimum allele depth) were reclassified as missing data following Dorant et al*.*^20^.

### Putative CNV identification

We used the *HDplot* method to detect duplicated SNPs representing putative CNVs from the above dataset following methods initially described by McKinney et al.^41^ and modified by Dorant et al*.*^20^. Putatively duplicated loci were identified using a combination of four parameters calculated for each locus: proportion of heterozygotes; inbreeding coefficient; median allele ratio for heterozygotes; and proportion of rare homozygotes. Each parameter was calculated using the *08_extract_snp_duplication_info.py* script downloaded from https://github.com/enormandeau/stacks_workflow. We plotted the four parameters against each other and used graphical cut-offs to categorize loci based on their position in the distribution using a modified *09_classify_snps.R* script downloaded from https://github.com/enormandeau/stacks_workflow. Putative CNVs were those loci categorized as duplicated, highly diverged, or had a high depth of coverage following Dorant et al*.*^20^ and Cayuela et al*.*^21^ (see Table S2 for summary of graphical cut-offs). We then extracted read depth for each putative CNV to use as a proxy for copy number^20,41^ using VCFtools *v*0.1.15^40^. To account for differences in sequencing effort across individuals, we normalized read counts using a trimmed mean of M-values method using the *edgeR* R-package^42^ following Dorant et al*.*^20^. Missing genotypes for an individual were imputed as the mean read depth for that locus across individuals within the same sample site.

### Genotype-environment association analyses

We performed several genotype-environment association (GEA) analyses to identify CNVs with putative links to climate adaptation. For this, we downloaded climate data for 27 variables from ClimateNA^43^ (Table S3) and separated them into three climate variable categories: temperature (*n* = 16); precipitation (*n* = 6); and solar radiation (*n* = 5). We separated variables into categories to assess the relative impact of temperature, precipitation, and solar radiation independently and to minimize redundancy across categories due to multicollinearity. To further reduce multicollinearity, we calculated correlation coefficients between each pair of climate variables within each variable category; for each pair with |*r|*> 0.70, we removed the variable with largest mean absolute correlation using the *findCorrelation* function as part of the *caret* R-package^44^.

We first used partial redundancy analysis (pRDA) to detect CNVs with climate associations separately for each climate variable category using the *vegan* R-package^45^. Climate variables were assigned as predictors with normalized read depth as the response. Due to sequencing batch effects detected in the normalized read depth matrices (Figure S1), we included the sequencing library ID for each individual as a covariate. To validate the absence of multicollinearity, we calculated the variance inflation factor (VIF) for each predictor variable and removed those with VIF > 10^46^. We assessed model significance using global and marginal analyses of variance (ANOVAs) with 1000 permutations and retained all significant (*p* ≤ 0.05) axes for downstream analysis. In the event of no significant axes, only the first RDA axis was retained for outlier detection. Climate-associated CNVs (i.e., outlier loci) were then classified as those with a loading > 2.25 standard deviations from the mean loading along each retained RDA axis (*p* ≤ 0.01)^47^.

We also used linear mixed-effect models (LMM) to detect climate-CNV associations for each climate variable category using the *lme4* R-package^48^. We used log-transformed normalized read depth as the response variable and the same climate variables as used for the pRDA as fixed effects; we included the sequencing library ID as a random intercept to account for batch effects. We assessed significance of each climate variable using the likelihood ratio test (LRT) as implemented by the *drop1* function in the *lmerTest* R-package^49^. To control for false positives, we corrected *p*-values using the Benjamini–Hochberg false discovery rate method with a corrected significance threshold of α = 0.05. As a final step to reduce false positives, we retained only those loci detected as outliers in both the LMM and pRDA and classified these as “robust outliers”. Finally, we re-ran the pRDAs using the methods above with only the robust outliers to estimate the amount of variation explained by the climate variables for these loci.

### Genetic differentiation and adaptive divergence

We estimated pairwise genetic differentiation using the variant fixation index *V*_ST_^50^ via a custom R function, which is analogous to the estimator of population differentiation *θ*^51^, and is commonly used to identify differentiated CNVs between populations^20,50^. Using the robust outliers for each climate category, *V*_ST_ estimates were calculated on a per locus basis and averaged across each pair of sites to obtain mean pairwise estimates. We assessed significance using a bootstrap resampling procedure implemented in the *boot* R-package^52^ for mean pairwise *V*_ST_ estimates across 10,000 replicates.

We also examined patterns of adaptive divergence within lineages using a hierarchal clustering approach. Using the robust outliers for each climate category, we generated a matrix of pairwise genetic distance by calculating Bray–Curtis distances for each pair of individuals using the *ecodist* R-package^53^ then calculated the mean distance for each pair of sites. Using this distance matrix as an input, we performed a hierarchal clustering analysis employing the Ward’s minimum variance method^54^ using the *hclust* R-function. The resulting dendrograms were bootstrapped with 10,000 replicates to assess robustness using *boot.phylo* function in the *ape* R-package^55^.

To assess the impact of geography on adaptive divergence, we performed linear regressions on each robust locus with either elevation or latitude as the explanatory variable and normalized read depth as the response. Linear regressions were performed in R using the base packages^56^.

### Annotation of putatively adaptive variants

To assess the putative functional implications of climate-associated CNVs, we annotated all robust outliers using the Ensembl Variant Effect Predictor *v*103.1^57^ and identified CNVs found within protein-coding genes. We then performed a literature search to explore the function of genes that had linked CNVs from across multiple lineages, were linked to multiple CNVs, or were linked to CNVs with significant associations to multiple climate variable categories.

## Results

### SNP identification and CNV detection

Initial SNP calling resulted in 359,569–1,170,051 loci genotyped per lineage (mean = 784,092 loci) with 60,383–150,978 loci remaining post-filtering (mean = 94,428 loci; Table S1). From the filtered datasets, we identified between 2208 and 9585 putative CNVs per lineage with a mean sequencing depth of 11.0x ± 3.62 SD (ranging from 4.3x  to 118.7x) for downstream analysis (Table S1). Mean missing data were 4% ± 4.2 SD per locus (range: 0–30%).

### GEA analyses and robust outliers

After removing colinear variables, we retained two to three variables within each climate variable category within each lineage (Table S4). For the pRDAs, all models were significant (*p* < 0.05) for all climate variable categories and all lineages except for the solar radiation model in the CU lineage (Table S4). Each RDA had at least one significant axis (*p* < 0.05) except for the CRM precipitation model (*p*_RDA1_ = 0.072), the SRM solar radiation model (*p*_RDA1_ = 0.120) and the CU solar radiation model (*p*_RDA1_ = 0.233); for these models, we still retained the first pRDA axis for outlier detection. The temperature model explained the most variation within each lineage (adjusted *r*^2^ = 0.018–0.064) except for SRM (adjusted *r*^2^ = 0.015), which had the precipitation model explaining the most variation (adjusted *r*^2^ = 0.017). We detected between 35 and 468 climate-associated CNVs for each of the pRDAs after removing duplicate loci (Table S4; Figs. S2–S7).

For the LMMs, we detected between 46 and 216 unique loci associated with at least one climate variable within each lineage (Tables S5–S10; Figs. S2–S7). We found the most significant associations with temperature for the NRM (79 unique loci), CSC (139 unique loci), and CU (116 unique loci) lineages, while precipitation had the most for the SRM (29 unique loci) and SN (112 unique loci) lineages. Solar radiation had the largest number of outlier loci for the CRM lineage (28 unique loci; Tables S5–S10; Fig. S2–S7).

**Figure 2:**
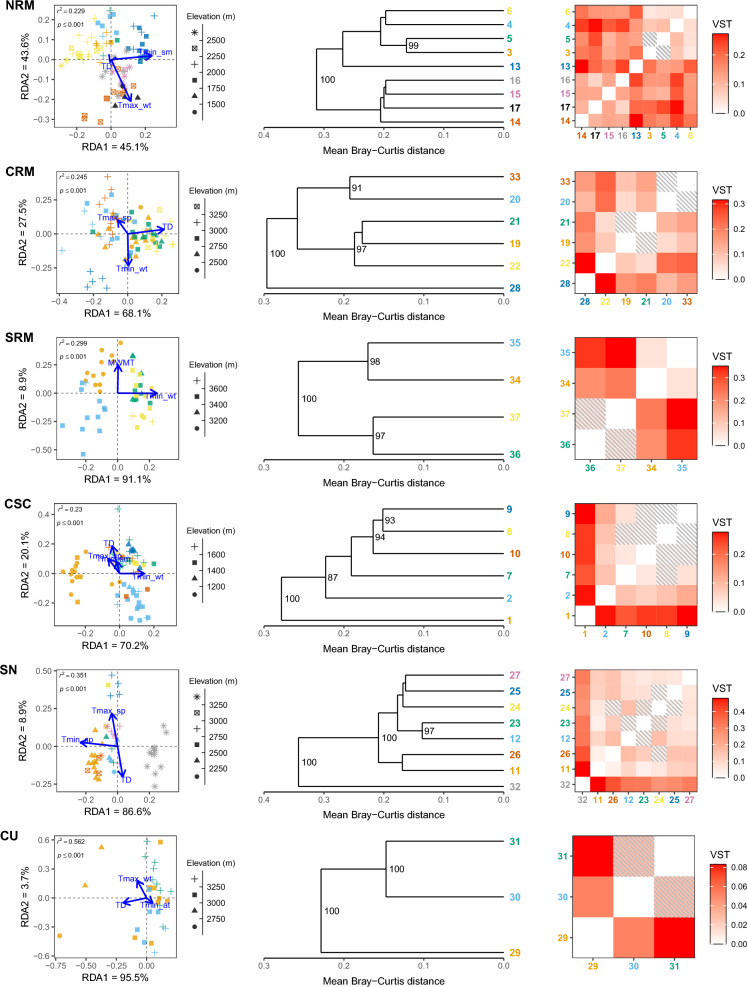
Adaptive divergence of robust temperature outliers in the American pika (*Ochotona princeps*). Partial redundancy analysis (left) was run using the *vegan* R-package^45^; sample sites are indicated by colour and correspond to the sample site numbers on the dendrograms (middle) and heatmaps (right). Sample site numbers correspond with those found on Fig. 1. Dendrograms (middle) were created by hierarchal clustering of Bray–Curtis distances with bootstrap values shown on the nodes; for clarity, only values > 80 are shown. Heatmaps (right) display pairwise population differentiation (*V*_ST_); shaded tiles indicate non-significant results from a bootstrapping resampling procedure with 10,000 replicates (*p* > 0.05). *NRM* Northern Rocky Mountains, *CRM* Central Rocky Mountains, *SRM* Southern Rocky Mountains, *CSC* Cascades, *SN* Sierra Nevada, *CU* Central Utah.

**Figure 3:**
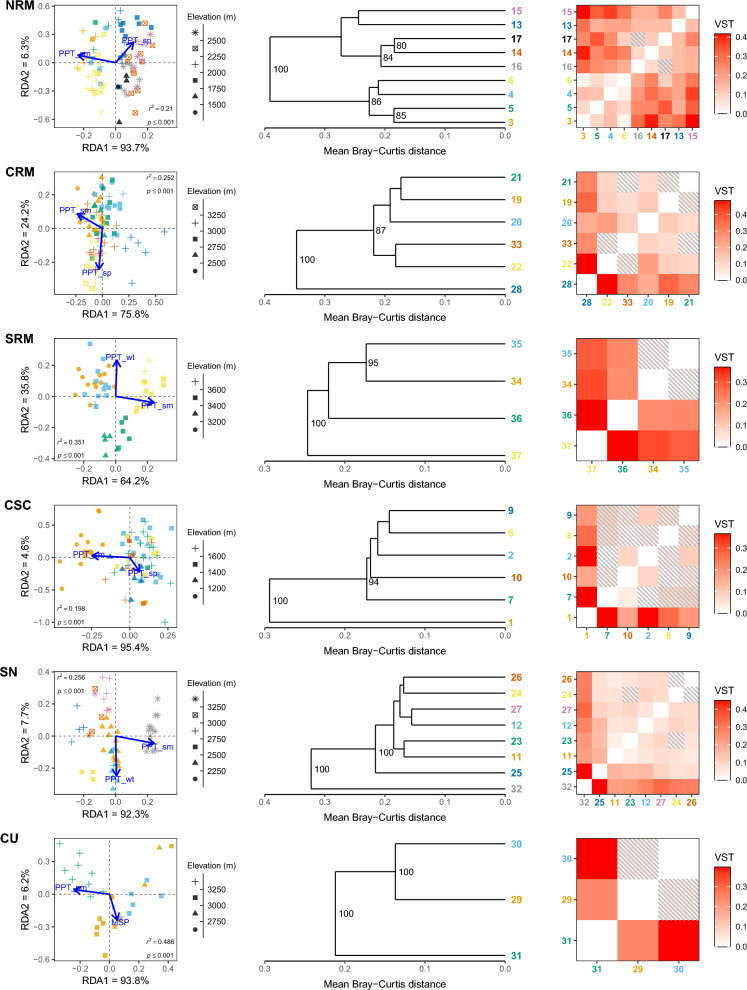
Adaptive divergence of robust precipitation outliers in the American pika (*Ochotona princeps*). Partial redundancy analysis (left) was run using the *vegan* R-package^45^; sample sites are indicated by colour and correspond to the sample site numbers on the dendrograms (middle) and heatmaps (right). Sample site numbers correspond with those found on Fig. 1. Dendrograms (middle) were created by hierarchal clustering of Bray–Curtis distances with bootstrap values shown on the nodes; for clarity, only values > 80 are shown. Heatmaps (right) display pairwise population differentiation (*V*_ST_); shaded tiles indicate non-significant results from a bootstrapping resampling procedure with 10,000 replicates (*p* > 0.05). *NRM* Northern Rocky Mountains, *CRM* Central Rocky Mountains, *SRM* Southern Rocky Mountains, *CSC* Cascades, *SN* Sierra Nevada, *CU* Central Utah.

**Figure 4:**
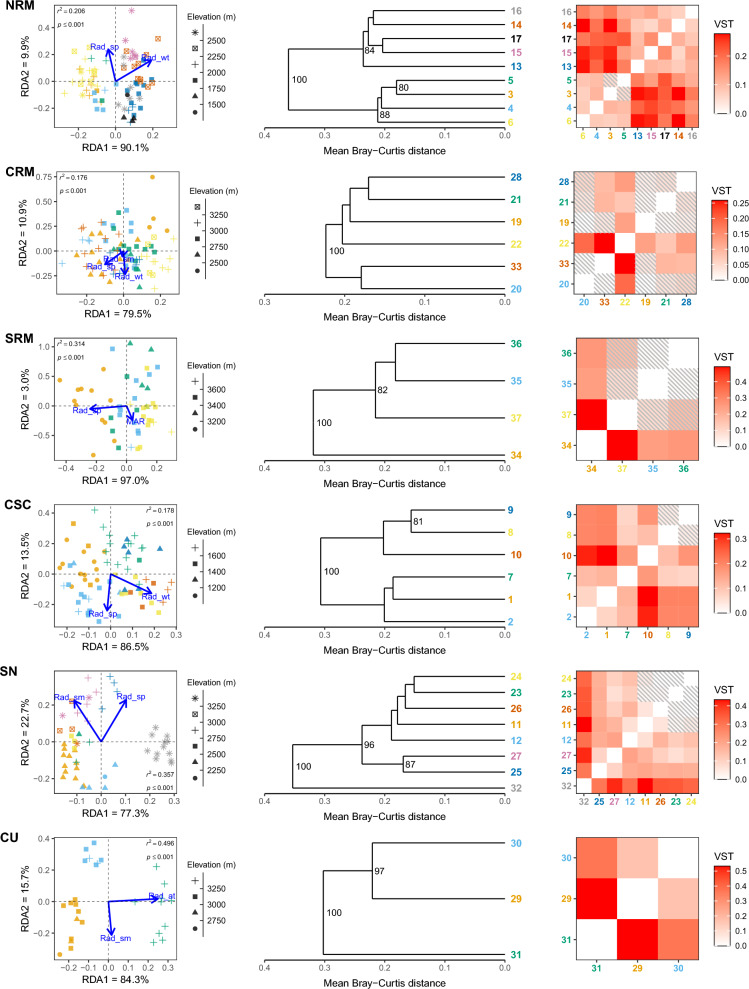
Adaptive divergence of robust solar radiation outliers in the American pika (*Ochotona princeps*). Partial redundancy analysis (left) was run using the *vegan* R-package^45^; sample sites are indicated by colour and correspond to the sample site numbers on the dendrograms (middle) and heatmaps (right). Sample site numbers correspond with those found on Fig. 1. Dendrograms (middle) were created by hierarchal clustering of Bray–Curtis distances with bootstrap values shown on the nodes; for clarity, only values > 80 are shown. Heatmaps (right) display pairwise population differentiation (*V*_ST_); shaded tiles indicate non-significant results from a bootstrapping resampling procedure with 10,000 replicates (*p* > 0.05). *NRM* Northern Rocky Mountains, *CRM* Central Rocky Mountains, *SRM* Southern Rocky Mountains, *CSC* Cascades, *SN* Sierra Nevada, *CU* Central Utah.

We found from 37 to 193 total robust outliers detected by both methods within each lineage (Figs. S2–S7). Repeating the pRDAs using only the robust outliers for each climate variable category resulted in substantially greater variation explained by climate (between 19.8 and 59.1%; Table 1; Figs. 2, 3, 4). All models were highly significant across all climate variable categories and lineages (*p* < 0.001; Table 1; Figs. 2, 3, 4).

**Table 1:** Summary values for redundancy analysis (RDA) and ANOVA performed on robust outlier loci for six American pika (*Ochotona princeps*) lineages.

Lineage	Category	*N*_robust_	*r*^2^	*r*^2^_adjusted_	*df*	*F*	*p*
NRM	Temperature	53	0.256	0.229	3	8.621	0.001
	Precipitation	26	0.228	0.210	2	11.175	0.001
	Solar radiation	39	0.224	0.206	2	10.777	0.001
CRM	Temperature	13	0.271	0.245	3	9.206	0.001
	Precipitation	9	0.267	0.252	2	14.163	0.001
	Solar radiation	15	0.205	0.176	3	6.506	0.001
SRM	Temperature	17	0.321	0.299	2	11.244	0.001
	Precipitation	23	0.370	0.351	2	14.044	0.001
	Solar radiation	8	0.337	0.314	2	11.489	0.001
CSC	Temperature	64	0.269	0.230	4	6.255	0.001
	Precipitation	7	0.217	0.198	2	9.548	0.001
	Solar radiation	31	0.198	0.178	2	8.732	0.001
SN	Temperature	62	0.383	0.351	3	9.834	0.001
	Precipitation	90	0.280	0.256	2	9.417	0.001
	Solar radiation	41	0.376	0.357	2	14.372	0.001
CU	Temperature	13	0.591	0.562	3	11.929	0.001
	Precipitation	33	0.507	0.486	2	12.807	0.001
	Solar radiation	90	0.515	0.496	2	13.845	0.001

Robust outliers (*N*_robust_) were those loci detected via RDA and linear mixed modelling.

### Genetic differentiation and adaptive divergence

**Figure 5:**
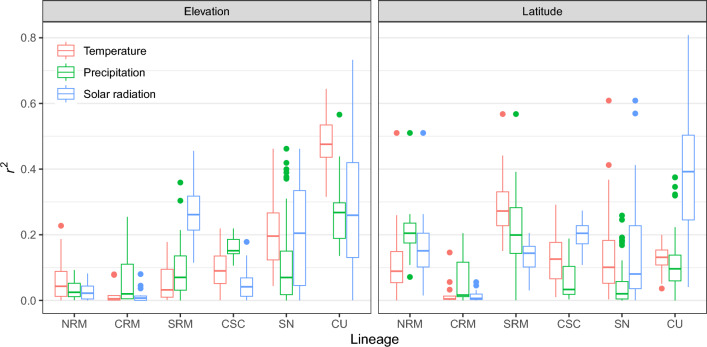
Boxplots showing the distribution of *r*^2^ values from linear regressions among robust climate-associated outlier loci in the American pika (*Ochotona princeps*). We performed independent linear regressions on each locus with either elevation or latitude as the explanatory variable and normalized read depth as the response. *NRM* Northern Rocky Mountains, *CRM* Central Rocky Mountains, *SRM* Southern Rocky Mountains, *CSC* Cascades, *SN* Sierra Nevada, *CU* Central Utah.

During preliminary analyses, we saw minimal to no evidence of population structure (Figure S1) and only weak population differentiation (all pairwise *V*_ST_ < 0.07) within each lineage using all putative CNVs. Using the robust outliers, we found consistent elevational and latitudinal patterns of population structure and population differentiation across analyses, though with slight differences between lineages and climate variable categories (Figs. 2, 3, 4, 5). Within the NRM lineage, we detected two main population clusters that largely followed latitudinal gradients (Figures S8–S10), though there were also weak clinal trends between normalized read depth of the top correlated outlier loci and elevation (Figures S11–S13). Population structure was less apparent within the CRM lineage, especially for the temperature (Fig. 2) and solar radiation (Fig. 4) outliers, though there were noticeable elevational patterns among precipitation outliers (Fig. S12). For the SRM lineage, we saw consistent patterns in population structure between temperature (Fig. 2) and precipitation (Fig. 3), with slightly different clustering for solar radiation (Fig. 4); however, we did see a distinct pattern between elevation (Figs. S11–S13) and latitude (Figures S8–S10) and normalized read depth. Temperature outliers seemed to be strongly correlated with elevation and latitude in the CSC lineage, while precipitation outliers seemed to be primarily associated with elevation, and radiation outliers followed latitudinal gradients (similar to temperature; Fig. 5). Structure within the SN lineage followed both elevational and latitudinal gradients for temperature and solar radiation outliers (Figs. 2, 4, 5); there was still distinct clustering by sample site using the precipitation outliers (Fig. 3), though there was no consistent pattern between either latitude or elevation and read depth (Figs. S9, S12). Population structure and differentiation most clearly followed elevational and latitudinal gradients in the CU lineage for all variable categories (Figs. 2, 3, 4, 5).

### Annotation of adaptive variants

We found that 207 of 508 unique robust outliers were located within introns, exons, or untranslated regions of protein-coding genes, with hits to 158 unique genes (Table S11). Temperature outliers had the most gene hits (*n* = 85 unique), followed by precipitation (*n* = 65 unique) and solar radiation (*n* = 55). Of these genes, 31 had associations across climate variables, 25 had associations across multiple CNVs, and five had associations across multiple lineages (Table S11). Additionally, one gene (*CHCHD3*) that had two associated CNVs was identified independently in both the NRM and CU lineages (Table 2, Table S11). Following our literature review, we identified 12 genes that had putative implications for local adaptation including those with functions regarding: mitochondrial structure and function (i.e., *CHCHD3*, *FBXL3*, *MCU*); immune response (i.e., *DOCK1*, *HPSE2, ONECUT1*); transcription (i.e., *MED12L*); hemoglobin structure and function (i.e., *HPSE2*); response to hypoxia (i.e., LOC101529014, *FBXL3*); olfaction (i.e., LOC101517538, LOC101532007); and DNA repair (i.e., LOC101529014, *HUS1*, *MACROD1*; Table 2).

**Table 2:** Subset of gene annotations for robust climate outliers detected using GEA analyses on six American pika (*Ochotona princeps*) lineages.

Gene ID	CNV	Variable associations	Lineages
*CHCHD3*	NC_050563.1_30967720_2611485	Radiation	CSC
	NC_050563.1_31126318_2611814	Temperature	NRM
	NC_050563.1_31126337_2611814	Temperature, precipitation, radiation	NRM, CU
	NC_050563.1_31126355_2611814	Temperature, precipitation, radiation	NRM, CU
	NC_050563.1_31126371_2611814	Precipitation, radiation	CU
	NC_050563.1_31126379_2611814	Precipitation, radiation	CU
*DOCK1*	NC_050553.1_3509915_140166	Precipitation	SRM
	NC_050553.1_3651078_140637	Precipitation, radiation	SN
*FBXL7*	NC_050546.1_30893897_950917	Temperature	CSC
	NC_050546.1_31094969_951406	Temperature	SN
	NC_050568.1_2668793_3906688	Temperature	CU
	NC_050568.1_2668814_3906688	Temperature	CU
*HPSE2*	NC_050553.1_25946746_201125	Precipitation	SN
	NC_050553.1_26022614_201248	Temperature	NRM
*HUS1*	NC_050545.1_42800208_3781296	Temperature, precipitation	SRM
LOC101517538 (*VMN2R116*)	NC_050553.1_69408172_305279	Temperature	NRM
	NC_050553.1_69408231_305279	Temperature	NRM
LOC101529014 (*TRRAP*)	NC_050548.1_133443909_2930290	Radiation	CU
	NC_050548.1_133443965_2930289	Radiation	CU
	NC_050548.1_133596957_2931039	Precipitation	CU
	NC_050548.1_133596959_2931039	Precipitation	CU
	NC_050548.1_37778753_2726048	Radiation	CU
	NC_050548.1_37779077_2726047	Radiation	CU
	NC_050548.1_37779097_2726047	Radiation	CU
	NC_050536.1_13032794_1943468	Temperature	CRM
	NC_050536.1_13033154_1943468	Temperature, radiation	CRM
	NC_050541.1_35683184_4832659	Precipitation	SN
	NC_050541.1_35703827_4832693	Radiation	CU
	NC_050541.1_35703843_4832693	Radiation	CU
	NC_050541.1_35703860_4832693	Radiation	CU
	NC_050541.1_35704145_4832693	Radiation	CU
	NC_050541.1_35704147_4832693	Radiation	CU
	NC_050541.1_35704162_4832693	Radiation	CU
	NC_050541.1_35704225_4832693	Radiation	CU
	NC_050542.1_9477885_1402332	Precipitation	NRM
	NC_050542.1_9477910_1402332	Precipitation	NRM
LOC101532007 (*OLFR147*)	NC_050536.1_13032794_1943468	Temperature	CRM
	NC_050536.1_13033154_1943468	Temperature, radiation	CRM
*MACROD1*	NC_050536.1_108586515_2151246	Temperature, precipitation	CSC
*MCU*	NC_050553.1_37066530_231578	Temperature, precipitation, radiation	SN
*MED12L*	NC_050549.1_14187010_4510924	Temperature	SN
	NC_050549.1_14187037_4510924	Temperature	SN
	NC_050549.1_14206971_4511008	Temperature	NRM
	NC_050539.1_2811551_2405314	Temperature	CSC
	NC_050539.1_2811556_2405314	Temperature	CSC
*ONECUT1*	NC_050552.1_65357252_2319361	Precipitation, radiation	CU

*NRM* Northern Rocky Mountains, *SRM* Southern Rocky Mountains, *CRM* Central Rocky Mountains, *CSC* Cascades, *SN* Sierra Nevada, *CU* Central Utah

## Discussion

### Structural variation and local adaptation

Various environmental factors can serve as drivers of local adaptation. For example, Muir et al*.*^58^ found that temperature significantly impacted larval period and growth rate in the common frog (*Rana temporaria*) distributed over an elevational gradient. Precipitation has been shown to be one of the most significant drivers of adaptation and natural selection on both continental and global scales^59,60^. High levels of solar (UV) radiation have led to rapid, convergent evolution of genes related to DNA repair in species residing on the Qinghai-Tibetan Plateau, the highest plateau on the planet^28^. Studying both the genetic and phenotypic impacts of environmental factors within and among species can improve understanding of biodiversity, speciation, and adaptation to heterogenous and changing landscapes.

There is a growing body of evidence that changes in genome structure may be an even more significant source of evolutionary potential than other, more well-studied markers such as SNPs^10,12,61–64^. Here, we found that structural variation in the form of copy number variation may be associated with local adaptation in the American pika. Specifically, we observed that putatively adaptive variation in this system largely followed elevational and latitudinal gradients. In the southwestern lineages (SN, CU), putatively adaptive variation was most strongly associated with elevational gradients, particularly with temperature (Figs. 2, 3, 4, 5). Populations of pikas in these regions are often limited to higher elevations, likely due to higher temperatures^25,26^; in fact, both recent and historical population declines have been documented in southwestern portions of the American pika range, primarily at lower elevations, and have been linked to warmer climates^65–68^. In the northern lineages (NRM, CSC), putatively adaptive variation closely followed latitudinal gradients, with elevational patterns also evident in the CSC lineage (Figs. 2, 3, 4, 5). Due to cooler temperatures at northern latitudes, pikas in these lineages can occupy a greater range of elevations and can be found as low as sea-level^69,70^. Additionally, the strength of latitudinal temperature gradients tends to increase moving northward from the tropics^71^ and could explain the difference in the effect of latitude between the northern and southern lineages. Furthermore, the northern lineages span a larger latitudinal gradient than the southern lineages, suggesting latitude has a greater potential to correlate with genetic variation in these regions. On the other hand, several studies have found that elevation plays a significant role in shaping putatively adaptive variation in northern populations of the American pika within the CSC lineage^31,32,72^, indicating that both spatial factors likely influence local adaptation in this system.

In contrast to the other southern lineages, we saw strong, latitudinal trends among outlier loci in the SRM lineage, though solar radiation in this lineage also strongly correlated with elevation (Fig. 5, Figs. S8–S10). Samples in this lineage were collected at high elevations (all > 3150 m) over a relatively small elevational gradient (~ 500 m difference between lowest and highest sites), which could explain the minimal impact of elevation in this lineage. The CRM lineage did not display any clear patterns and had significantly less structure when compared to the other lineages (Figs. 2, 3, 4, 5), possibly due to a narrow and intermediate latitudinal distribution that could limit differences in climate among sample sites. Alternatively, the genetic variation resulting from the relatively disjunct distribution between sample sites in this lineage, particularly in the case of site 28, may be masking signals of climate adaptation. This lineage is also the most recently diverged of the six phylogroups^29^ and may have had insufficient time post-divergence for selection to leave a significant signature of adaptive variation. Nevertheless, these patterns highlight the importance in sampling over appropriate environmental gradients to detect local adaptation^73^.

### Potential relationships between CNVs, gene expression, and phenotype

Copy number variation can have significant phenotypic and adaptive consequences^22,74–76^. CNVs are the most abundant form of structural variation, accounting for up to 10% of the total length of the human genome. Moreover, they even occur more frequently than SNPs^50,77–79^ and can serve as a significant source of selectable material, particularly when genes are located within a CNV^10,12,14,15^. CNVs can directly alter gene expression through increases/decreases in copy number, cause the inactivation of genes via duplication, and lead to amino acid changes and/or reading frame shifts when genes are only partially covered by a variant. In fact, CNV-linked genes more often have functions related to environmental response compared to basic cellular processes^74^. Copy number variation can also lead to high elevation adaptation. A study involving five species of domestic livestock found that mtDNA copy number decreased among high elevation populations compared to low elevation populations, which was hypothesized to be the result of chronic hypoxia^80^. Copy number variation has also been linked to high elevation adaptation in a number of other species, including yak^81^, ground tit^82^, and humans^83^. Further investigations of CNVs and high elevation environments may lead to a more thorough understanding of adaptations to hypoxia, cold stress, and UV radiation.

We found numerous CNVs within genes with putative links to climate and high elevation adaptation related to mitochondrial function, response to hypoxia, and DNA repair. *CHCHD3* had the greatest support for being under selection of all the annotated genes, as it was detected across all variable categories, several lineages (NRM, CSC, and CU), and multiple CNVs (*n* = 6; Table 2). This gene is critical in the formation of mitochondrial cristae. In fact, in vitro knockdown of *CHCHD3* results in significantly reduced oxygen consumption and glycolytic rates^84,85^, indicating that this gene could have potential consequences for cold tolerance and adaptation to hypoxia. *FBXL7*, another gene identified in our study as a potential target of selection, is also an important regulator of mitochondrial function. Expression levels of *FBXL7* were down-regulated under hypoxic conditions in the marine medaka^86^, and constitute an important predictor of the severity of asthma symptoms in humans^87^. We also found one CNV annotated to the mitochondrial calcium uniport (*MCU*), which is an integral component of the mitochondrial inner membrane^88^. These results are consistent with previous work that identified functional enrichment and positively selected genes associated with mitochondrial structure and function in the American pika reference genome^33,34^, providing further evidence for adaptation to high elevation environments in this species.

In addition, we found several genes associated with hypoxia response to be potentially under selection. The gene *HPSE2* encodes for the enzyme heparanase-2, which plays a role in extracellular matrix remodelling as well as embryo implantation. *HPSE2* has also been linked to hemoglobin-related traits, including fetal hemoglobin in North African human populations, which could have potential implications for adaptation to hypoxia^89,90^. We found that a predicted *TRRAP* ortholog (LOC101529014) also may be under selection. This gene is part of the *INO80* family of chromatin remodelers, which appear to have putative links to the response to hypoxia by interacting with hypoxia inducible factor-1^91,92^.

We further found that several genes related to DNA repair may be under selection. For example, *TRRAP* (discussed above) is also involved in DNA repair by binding with the *MRN*-complex to detect and repair double-strand breaks (DSBs)^93,94^. Knockout and knockdown of *TRRAP* results in the reduced efficiency and precision of end-joining following DSBs in mice and HeLa cells, suggesting this gene plays an important role in DSB signalling and repair^94^. The gene *HUS1* is part of the Rad9-Hus1-Rad1 complex, an important component of the DNA repair pathway. This complex loads onto damaged chromatin (for example, from UV exposure), promoting DSB repair^95^. Again, these results are consistent with previous studies showing putative adaptation to increased UV radiation at high elevations in American pika^33,34^.

### Other putatively adaptive genes

We found several other genes with putative links to local adaptation in the American pika. For example, the gene *MED12L—*a transcriptional coactivator of RNA poly II-dependent genes—was significantly associated with temperature in the NRM, CSC, and SN lineages (Table 2), and has been linked to elevational gradients in North American deer mice^96^ as well as mean annual temperature in Mediterranean cattle breeds^97^. We also found two genes, LOC101532007 (olfactory receptor 147-like) and LOC101517538 (vomeronasal type-2 receptor 116-like), which encode for olfactory receptors that could have an impact on foraging. American pikas do not hibernate; rather, they remain active throughout the winter and cache food into “hay piles” to ensure adequate food supplies^98^. These hay piles often consist of the highest quality vegetation available, likely detected and assessed via olfaction^99^; additionally, many of these cached foods contain high levels of secondary compounds that can help preserve biomass and nutrient availability into the winter months^100^. Differences in vegetation quality could also be linked to variation in precipitation, though the CNVs detected here were significantly associated with temperature. Evidence for putative adaptations related to olfaction has been previously shown in the American pika genome^34^.

We found further evidence for adaptations related to immune response. *DOCK1* is a gene required for phagocytosis of apoptotic cells and has been linked to immune response and climate adaptation in Middle Eastern sheep^101^ and disease resistance in dolphins^102^. *HPSE2* (discussed above) is also involved in the immune response, and expression levels of this gene were significantly associated with white blood cell count in pigs^103^. Similarly, *ONECUT1* is involved in B cell differentiation and has been linked to local adaptation in Ethiopian cattle^104^ and three-spined stickleback^105^. American pikas experience relatively high levels of parasitism and may be experiencing a spillover of parasites from other small mammals, particularly rodents, which could result in immune response adaptations^106–108^. Additionally, some populations of American pikas may be physiologically stressed due to harsh environmental conditions^109–113^; enhanced immune response may confer a greater ability for this species to survive and thrive.

### Limitations and future directions

Although we identified putatively adaptative variation in CNVs within the American pika, there were limitations to the approach employed in this study. We used climate data downloaded from ClimateNA which comes with several considerations^43^. First, this dataset uses climate data collected from 4891 weather stations distributed throughout North America to interpolate climate variables for any given set of coordinates, adjusting for elevation^43^. While this method allows for the estimation of climate data anywhere on the continent without requiring direct sampling, it also introduces potential error to climate variables. Wang et al*.*^43^ found that temperature estimates using this method generally correlated with direct measurements, whereas precipitation varied considerably more; additionally, elevation affected the accuracy of climate variables, with mountainous regions having lower accuracy in point estimates compared to flat regions. Second, this dataset measures total solar radiation of which harmful UV radiation only accounts for a small percentage (~ 5%)^114,115^. The relative contribution of UV radiation to total solar radiation also varies with elevation. However, both total solar radiation and UV radiation increase with elevation; as such, total solar radiation could operate as a crude proxy for UV radiation in lieu of a better estimate. These limitations could explain why both precipitation and solar radiation were relatively less correlated with copy number variation than temperature in this study. Lastly, these data only include ambient temperatures and do not account for the microclimates found underneath the talus that American pikas use for behavioural thermoregulation to prevent over-heating^116^. Recent findings suggest that subsurface microclimates important for pika thermoregulation may have changed at a faster rate than ambient temperatures over the past few decades in the Southern Rocky Mountains^117^. Consequently, the sole reliance on ambient temperature estimates may mask potential associations between CNVs and microclimate variation, which represents a potentially interesting avenue for future inquiry.

Our initial study design included plans to detect CNVs using a range-wide dataset. To take advantage of existing sequencing data, we used the *HDplot* method to detect CNVs^13,20,41^. This method visually detects putative paralogous loci by plotting several heterozygosity and genetic diversity metrics, and qualitatively identifies those that deviate from a central distribution. However, American pikas generally have low heterozygosity as a species at a range-wide level^38,39^, meaning standard SNP filtering procedures remove many variants. We found that the variants that remained showed very low levels of polymorphism with minimal deviations from the central distribution, greatly inhibiting our ability to confidently and accurately call putative CNVs using the *HDplot* method. To improve upon the ability to detect CNVs on a range-wide rather than within lineage scale, future work could employ whole genome resequencing that would provide a greater breadth of coverage and offer the added capability of detecting additional classes of structural variants, such as inversions and translocations^118^ that may be more directly associated with climate adaptation in the American pika.

## Conclusions

Here, we present a novel analysis of local adaptation in the American pika based on copy number variation. We found that CNVs were significantly associated with temperature, precipitation, and solar radiation within each lineage, and trends in putatively adaptive variation largely followed elevational and latitudinal gradients. Additionally, we identified several genes related to putative high elevation and climate adaptation that could serve as important targets in future studies, including those explicitly involving gene expression. Overall, our work adds to a growing body of literature revealing the novel insights that may be obtained by explicitly examining structural variation in the genome for investigating species-level responses to changing environments^20,119^. With climate change significantly altering habitats worldwide, a fuller understanding of how organisms may respond, including sentinel species such as the American pika^65,112^, will be critical for maintaining global biodiversity moving forward.

### Supplementary Information

Supplementary Figure S1.

Supplementary Figure S2.

Supplementary Figure S3.

Supplementary Figure S4.

Supplementary Figure S5.

Supplementary Figure S6.

Supplementary Figure S7.

Supplementary Figure S8.

Supplementary Figure S9.

Supplementary Figure S10.

Supplementary Figure S11.

Supplementary Figure S12.

Supplementary Figure S13.

Supplementary Legends.

Supplementary Tables.

## Data Availability

Previously archived sequencing data are available from the NCBI sequence read archive (BioProject ID: PRJNA1075342). Normalized read depth for identified CNVs and SNP genotypic data are available in DRYAD (10.5061/dryad.2bvq83bzf). Climate data are available in the Supplementary Tables document (Table S3). Scripts used for CNV detection are publicly available at https://github.com/enormandeau/stacks_workflow. Benefits from this research accrue from the sharing of our data and results within public databases as described above.
