# Supplementary figures and images for: Putative climate adaptation in American pikas (Ochotona princeps) is associated with copy number variation across environmental gradients
Source: Sci Rep. 2024 Apr 13;14:8568. doi: 10.1038/s41598-024-59157-6 (PMC11014952; doi:10.1038/s41598-024-59157-6)

NRM

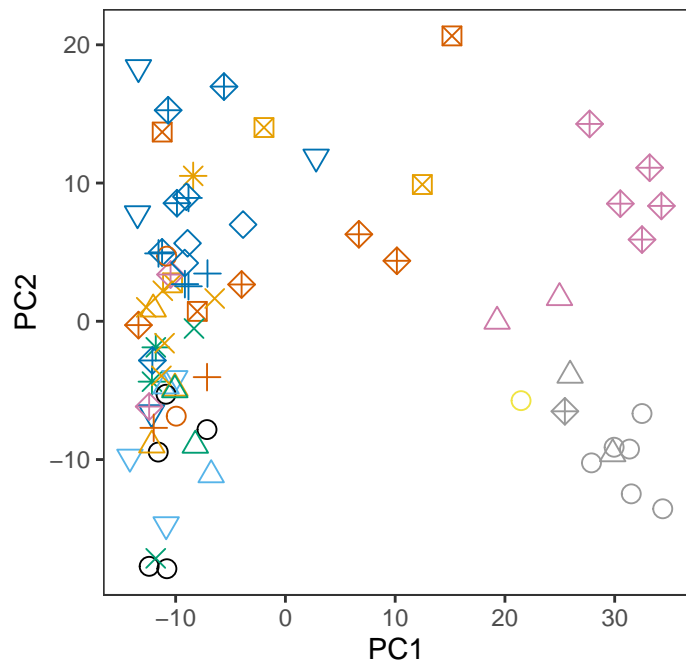

CRM

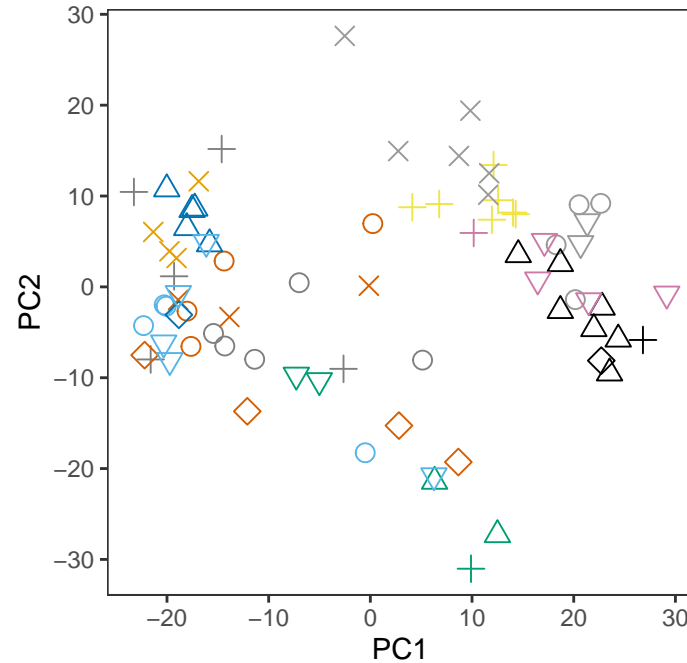

SRM

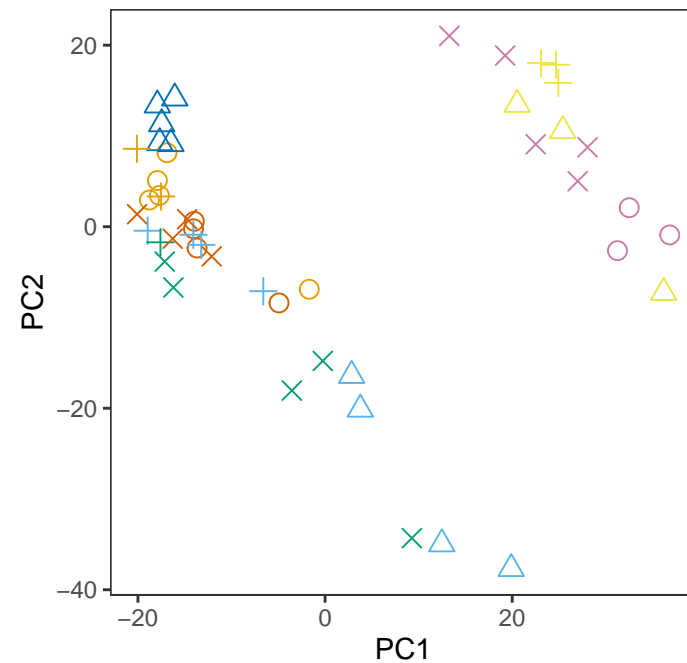

CSC

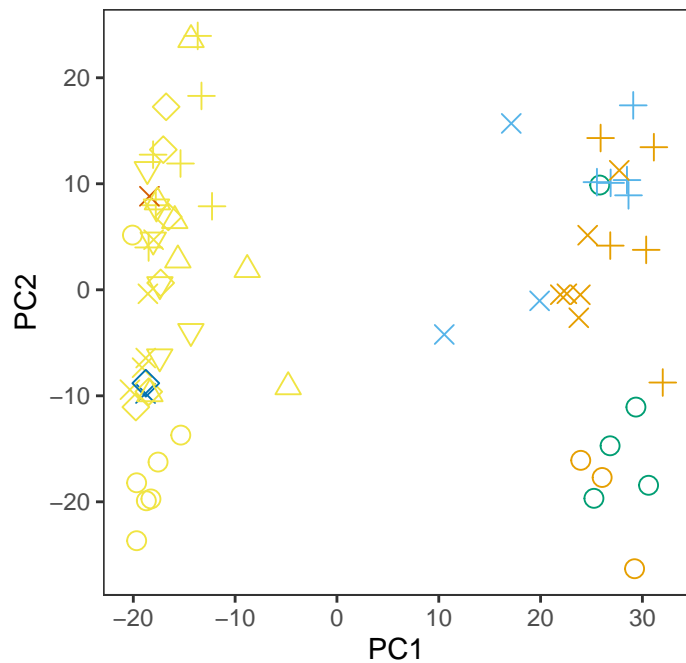

SN

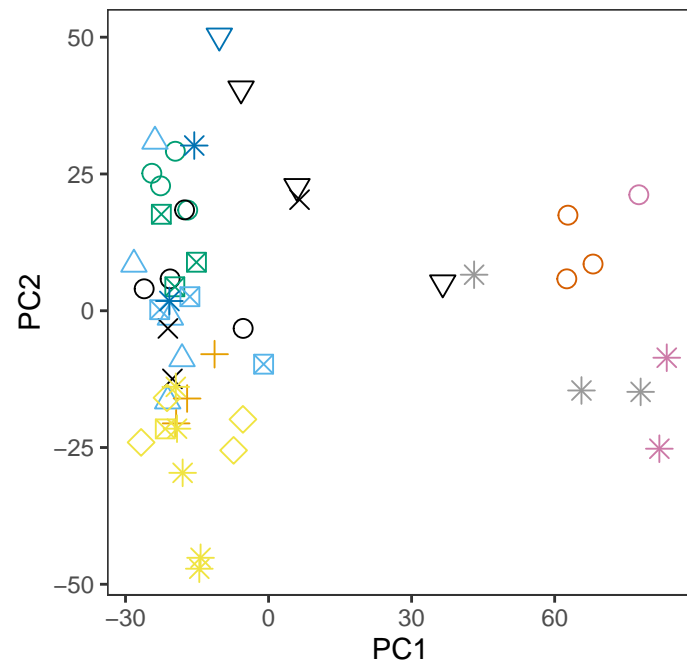

CU

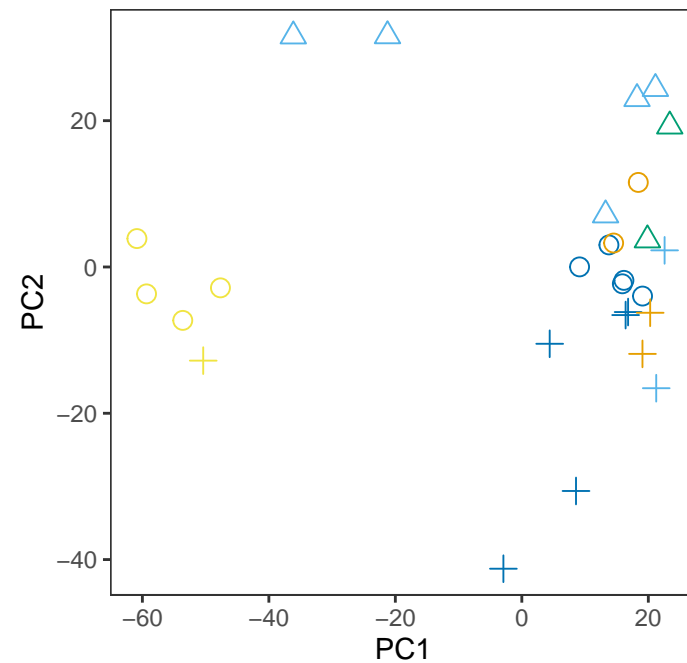

Supplement: Supplementary file 1 — Supplementary Figure S1. [file 41598_2024_59157_MOESM1_ESM.pdf]

# NRM – 2210 CNVs

Temperature

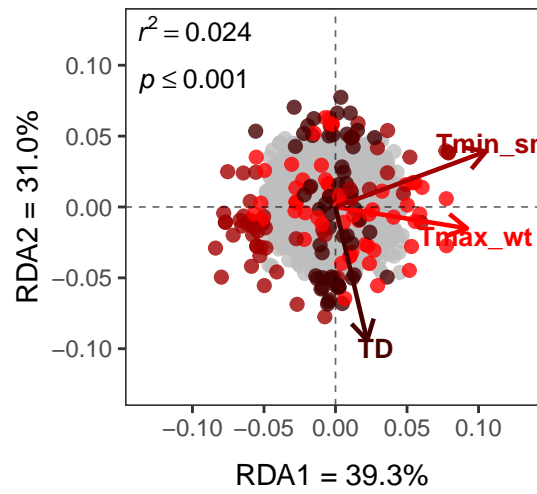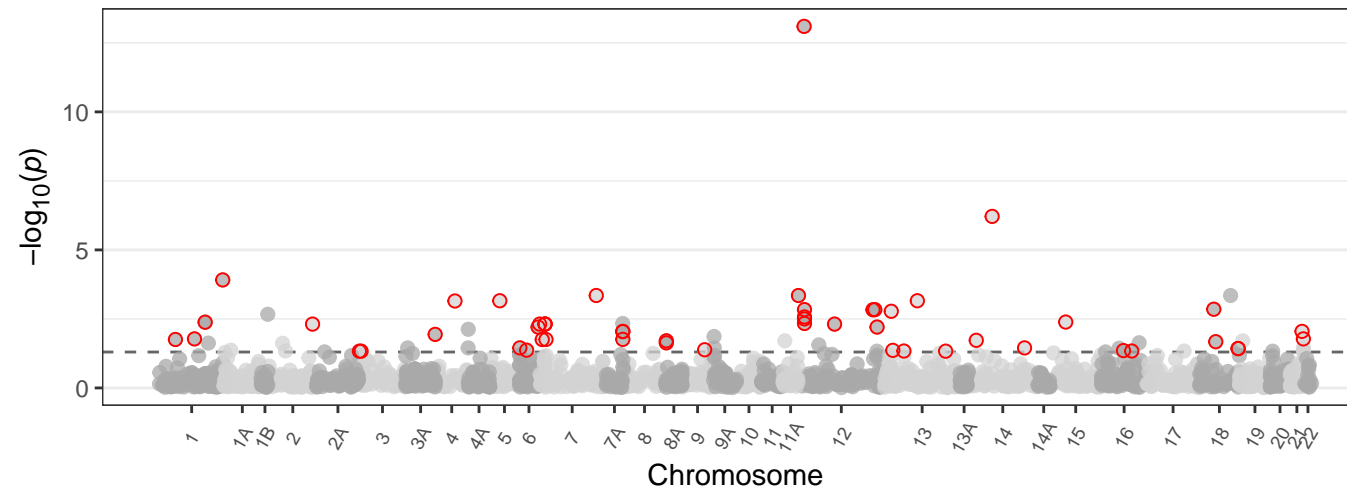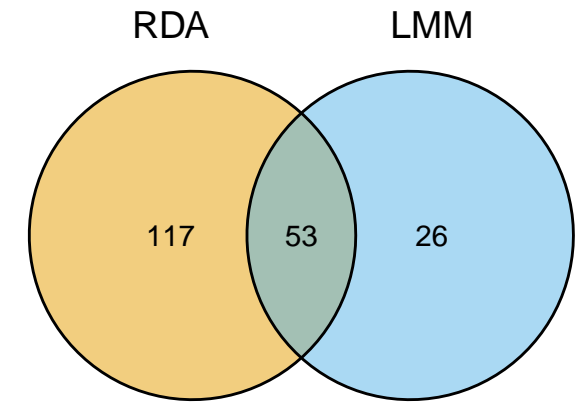

Precipitation

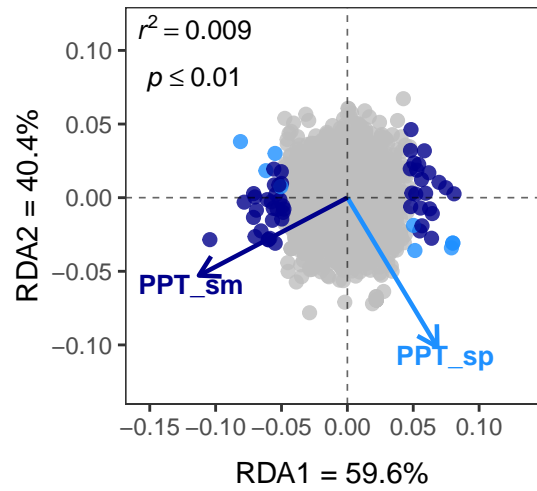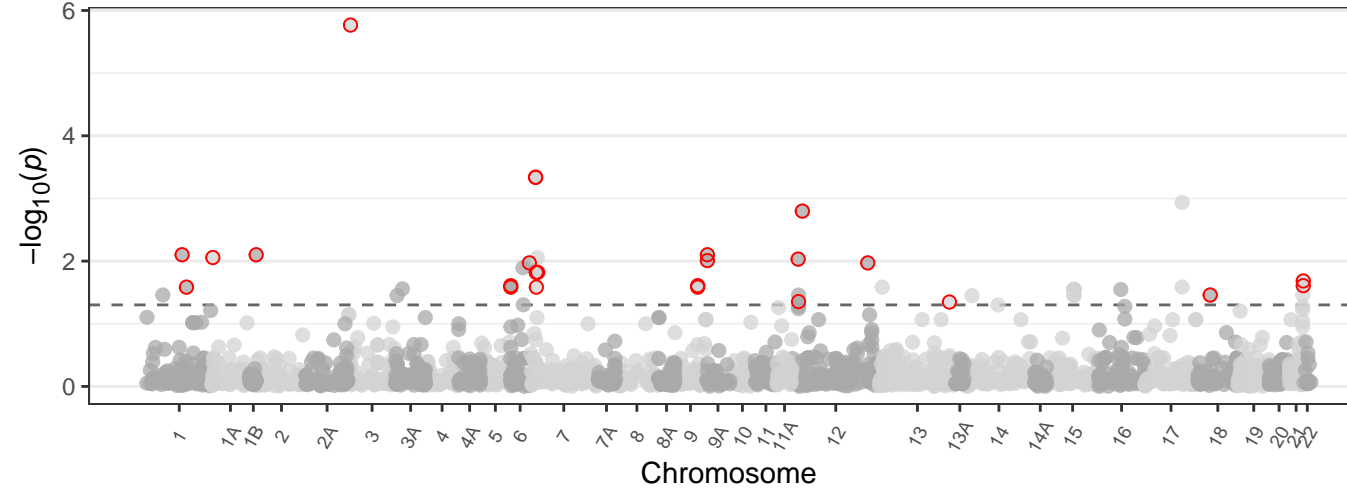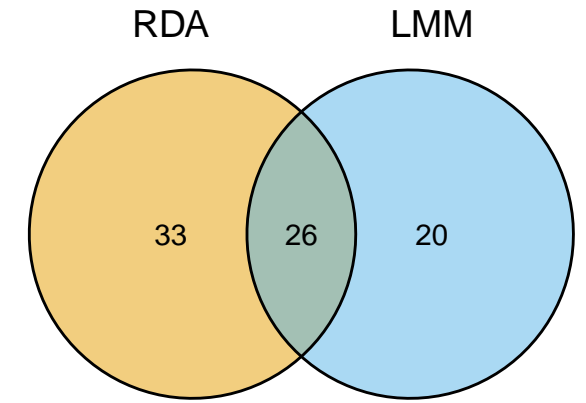

Solar radiation

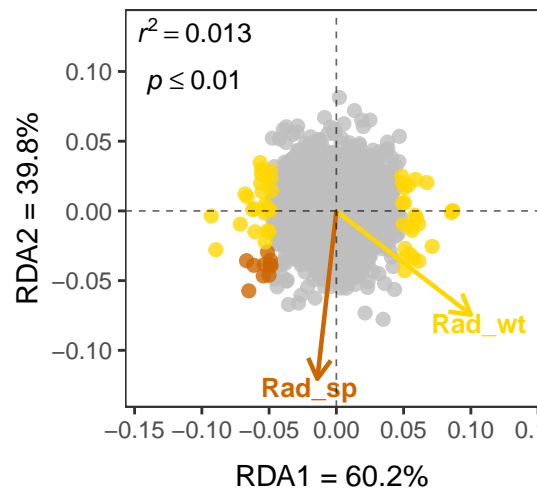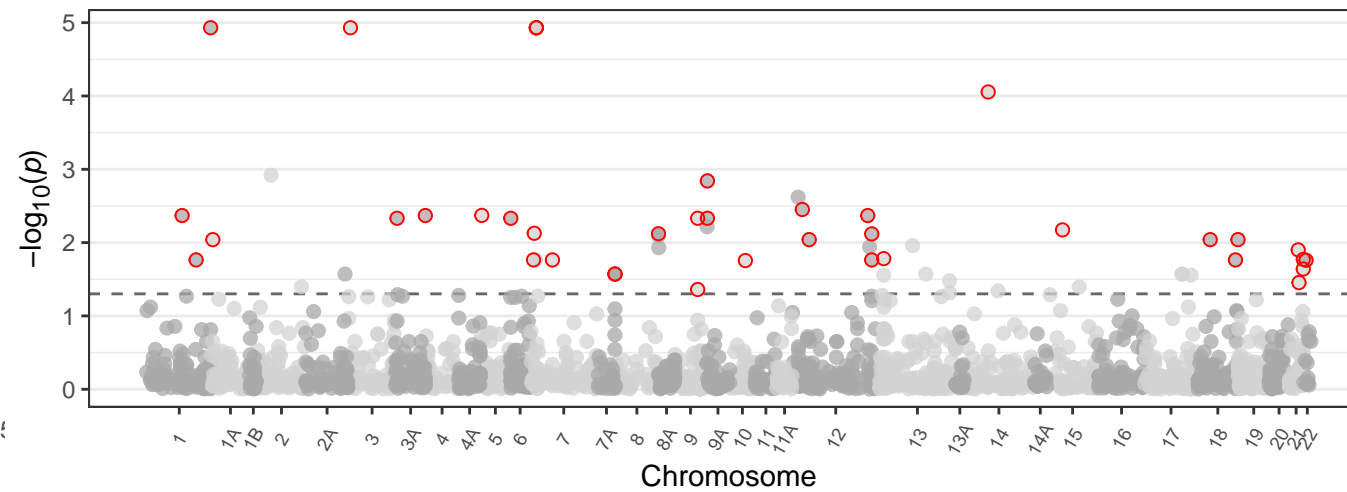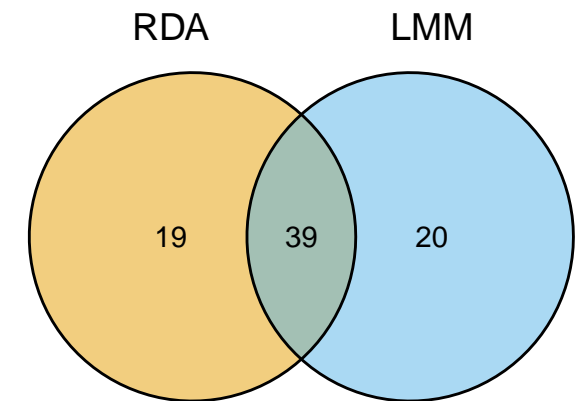

Supplement: Supplementary file 2 — Supplementary Figure S2. [file 41598_2024_59157_MOESM2_ESM.pdf]

# CRM – 2731 CNVs

Temperature

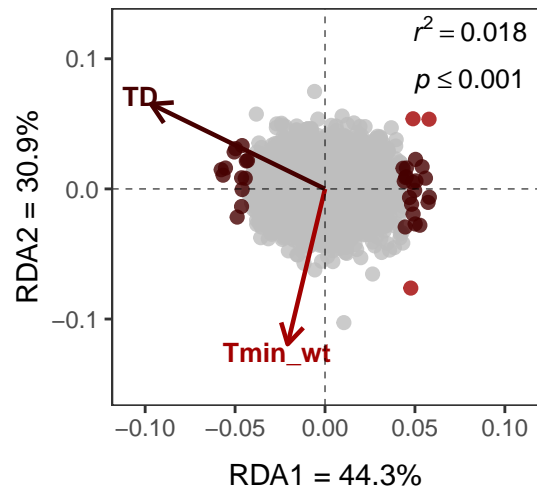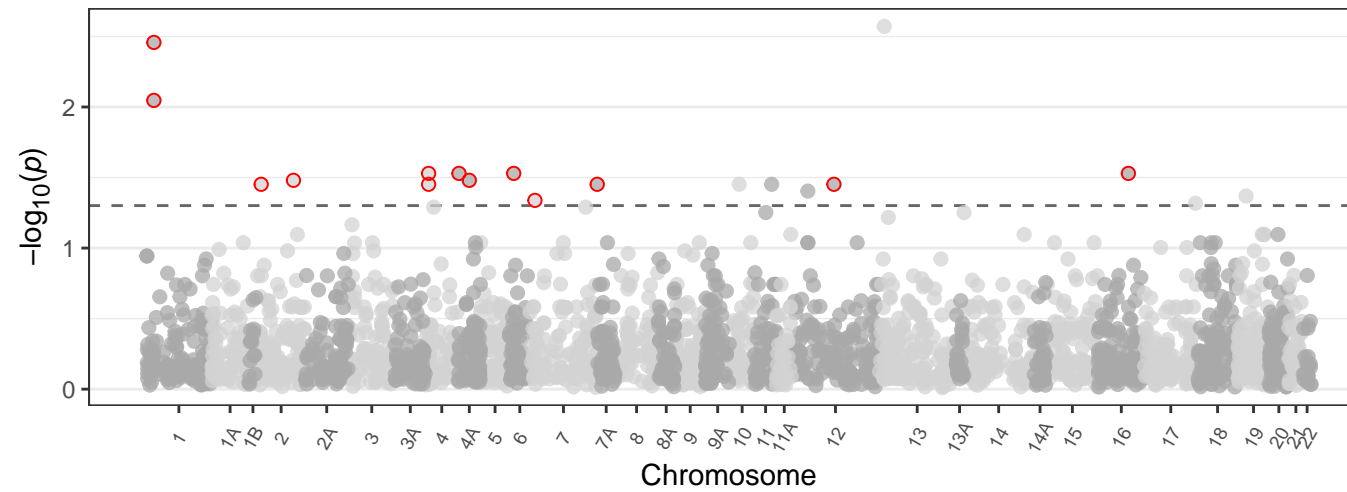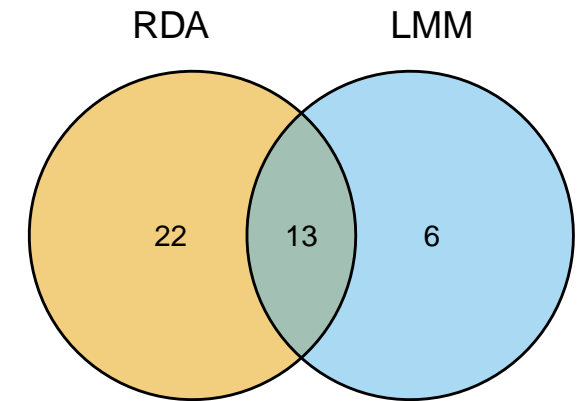

Precipitation

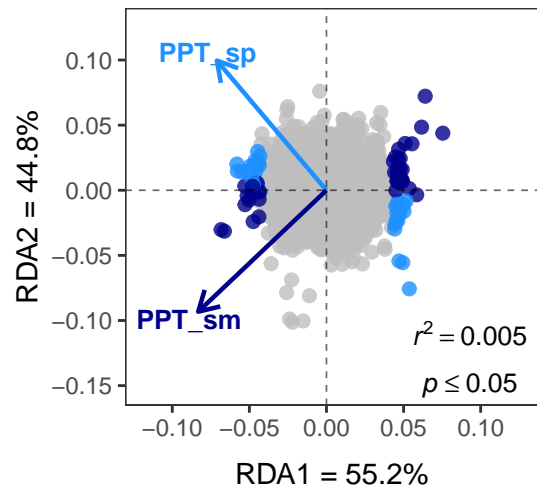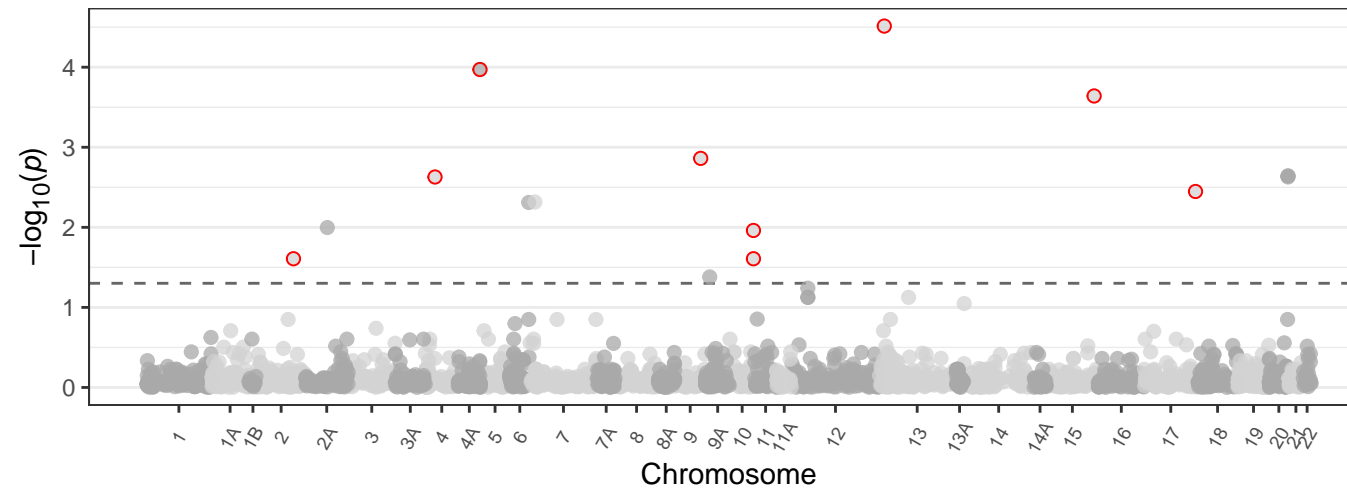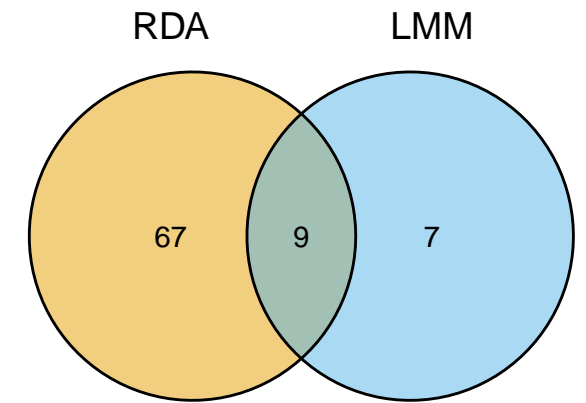

Solar radiation

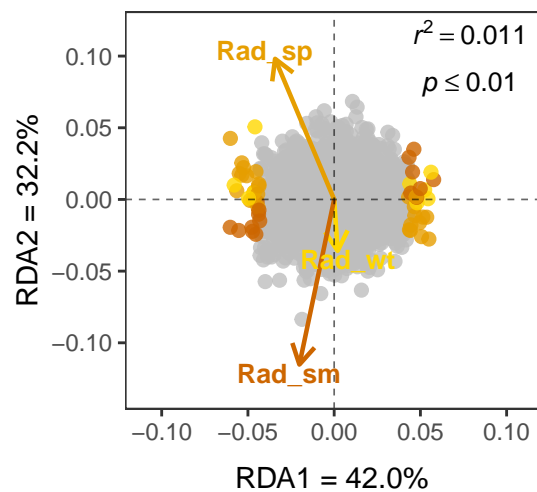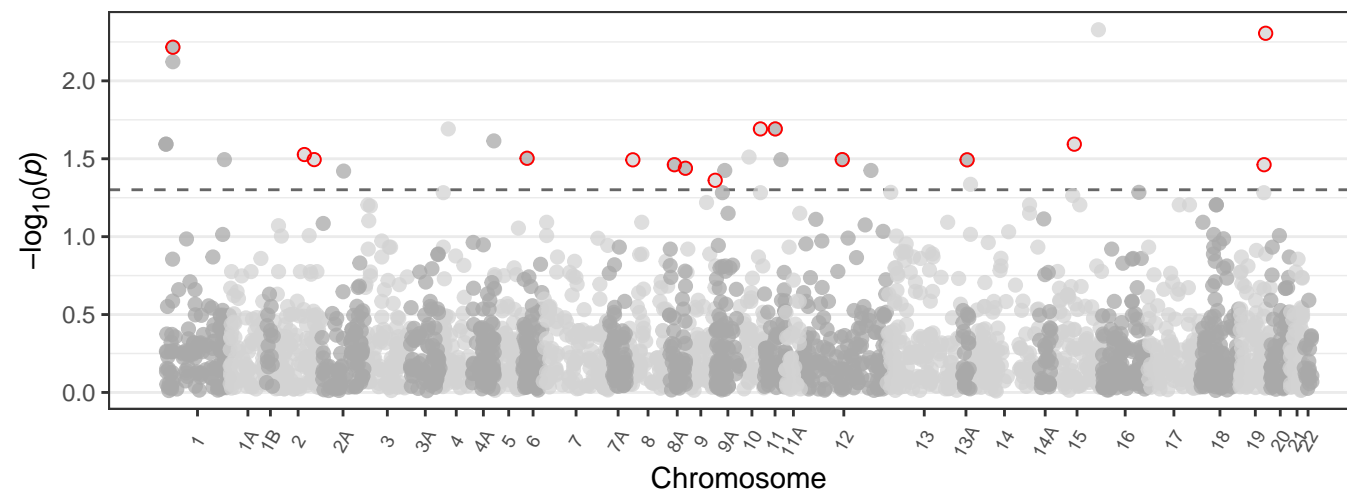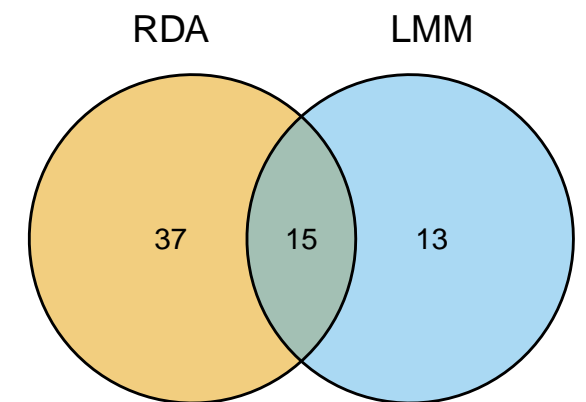

Supplement: Supplementary file 3 — Supplementary Figure S3. [file 41598_2024_59157_MOESM3_ESM.pdf]

# SRM – 3552 CNVs

Temperature

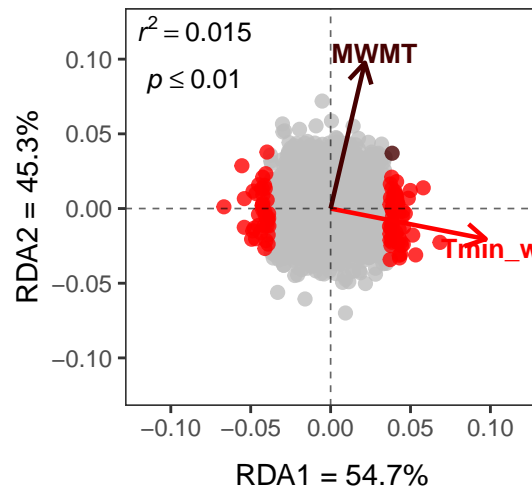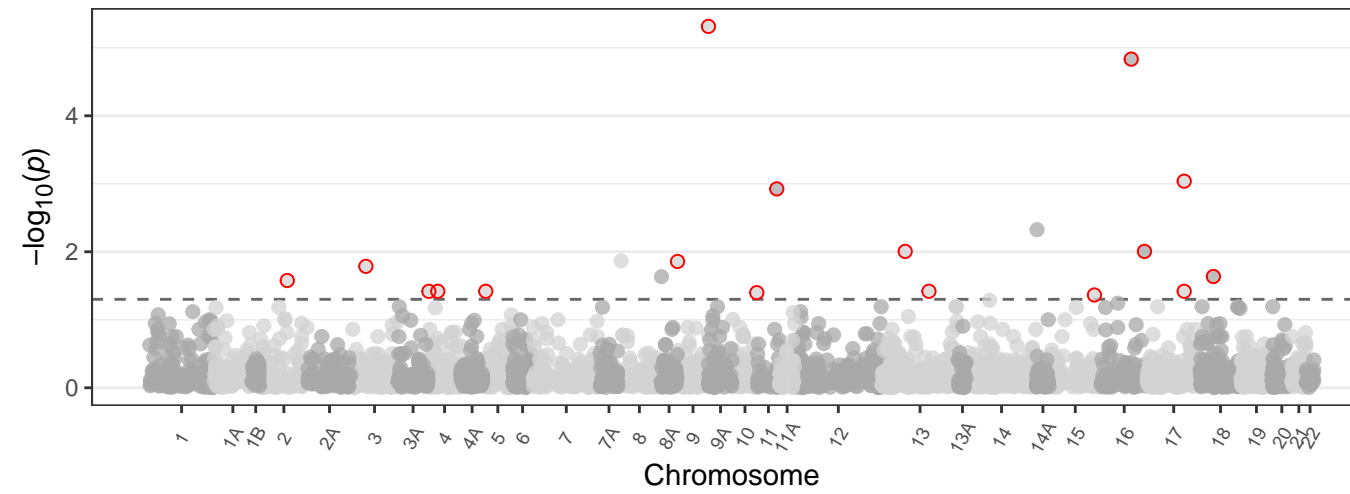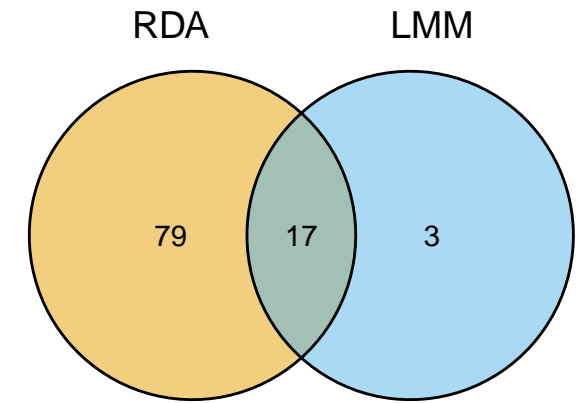

Precipitation

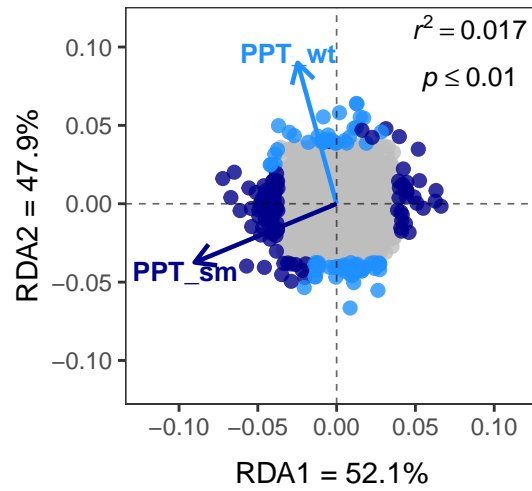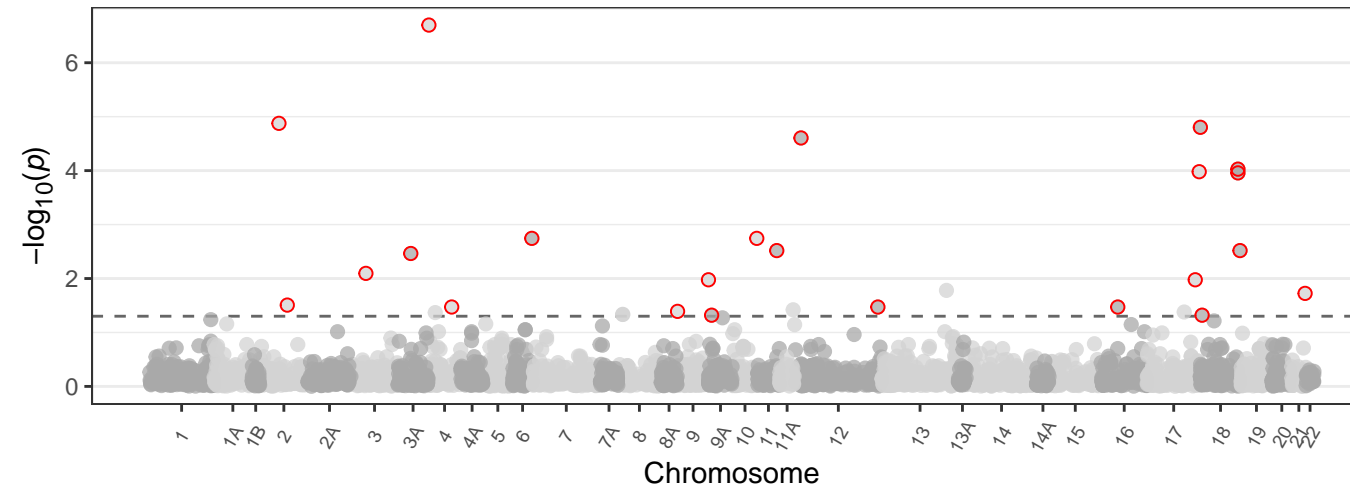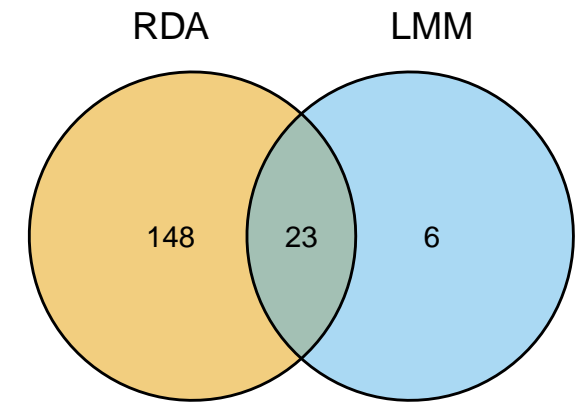

Solar radiation

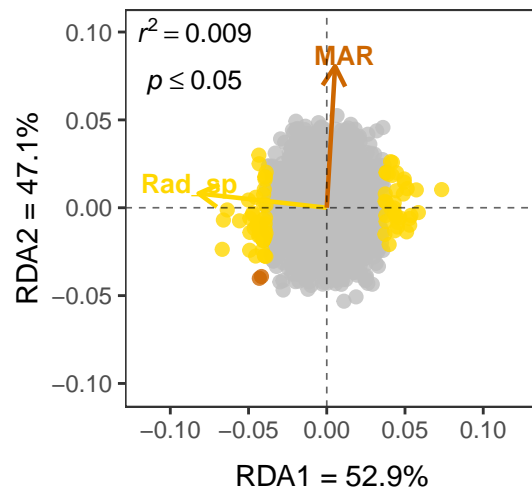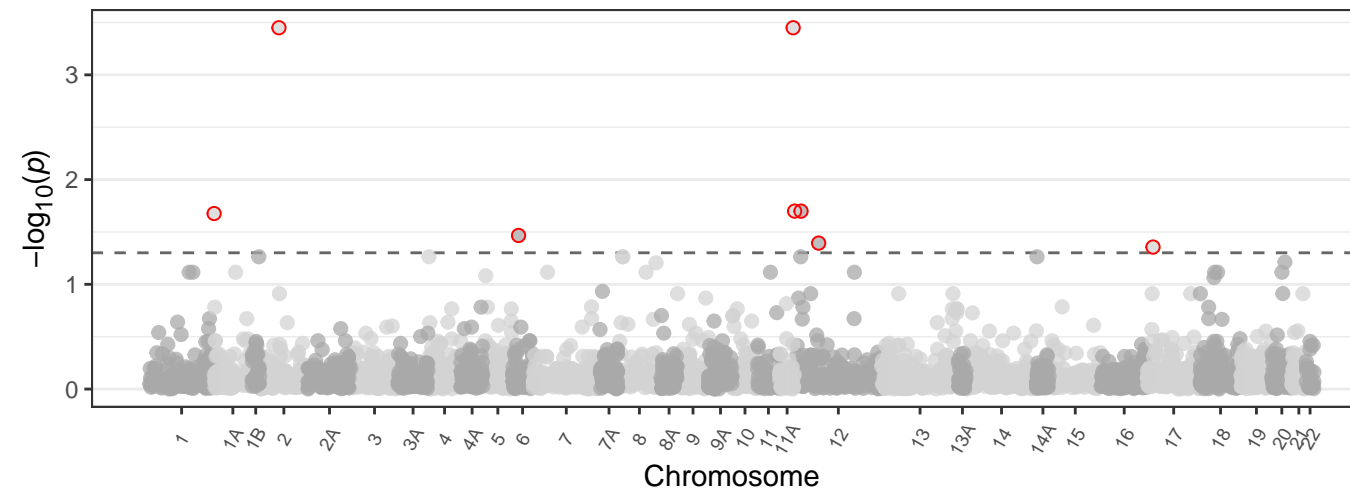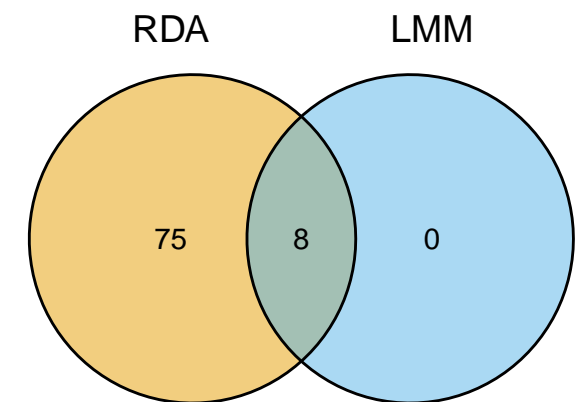

Supplement: Supplementary file 4 — Supplementary Figure S4. [file 41598_2024_59157_MOESM4_ESM.pdf]

# CSC – 3094 CNVs

Temperature

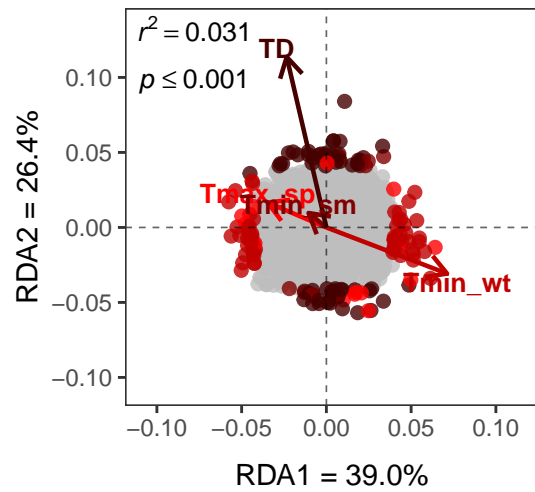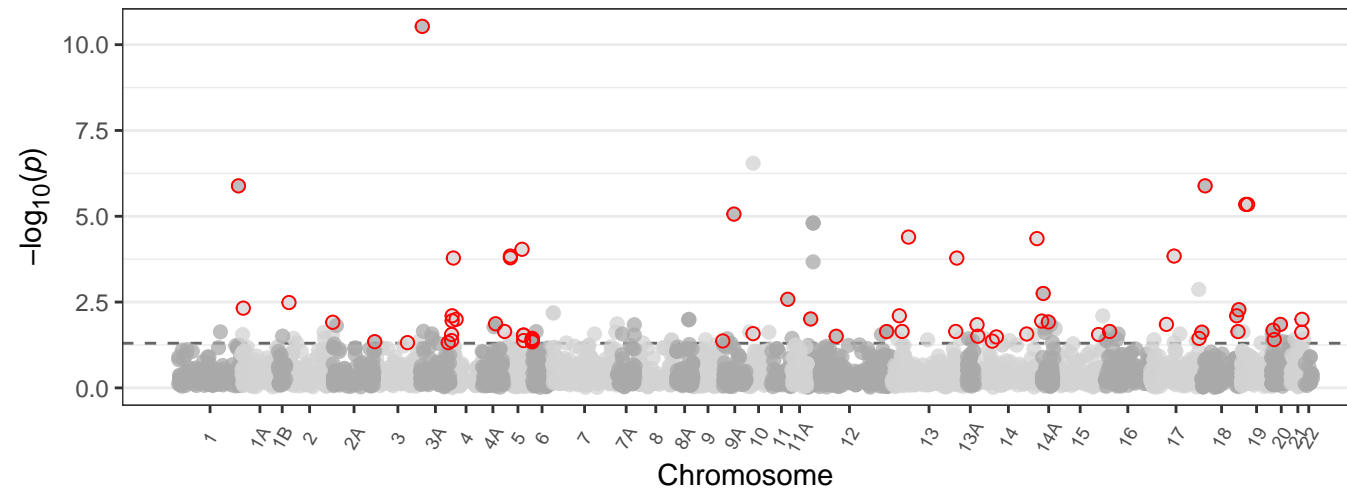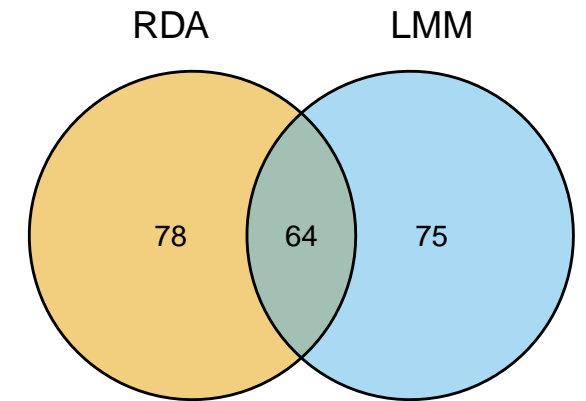

Precipitation

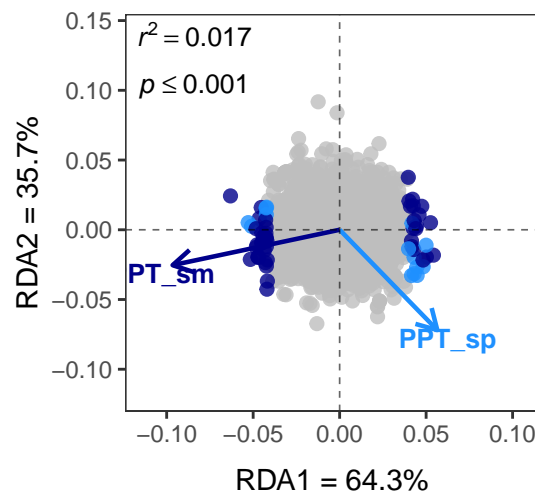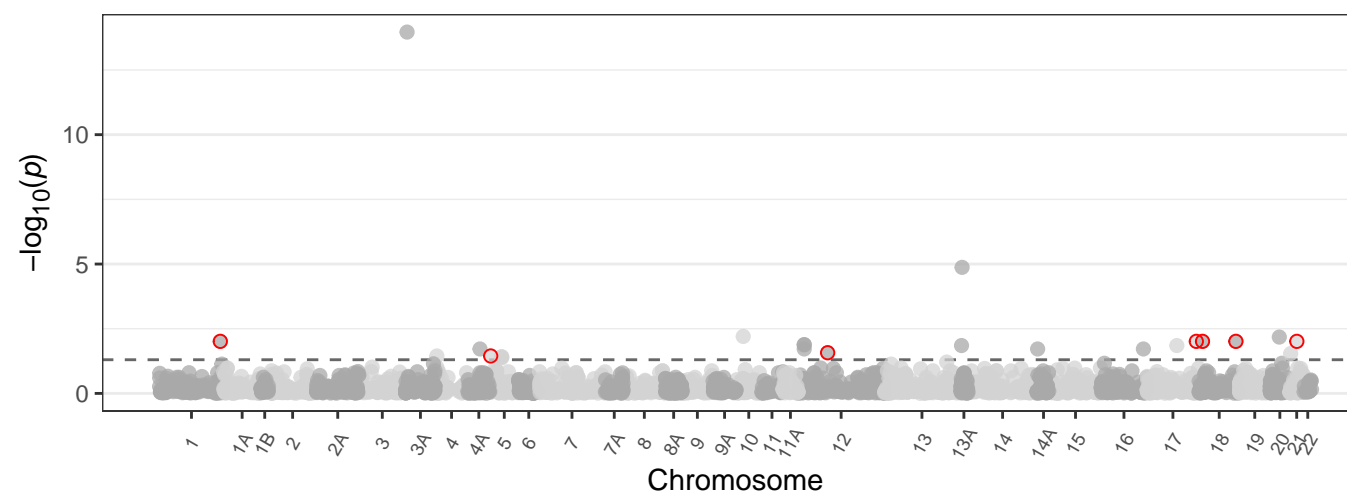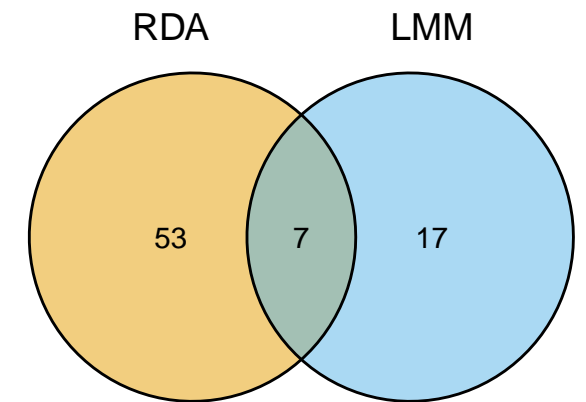

Solar radiation

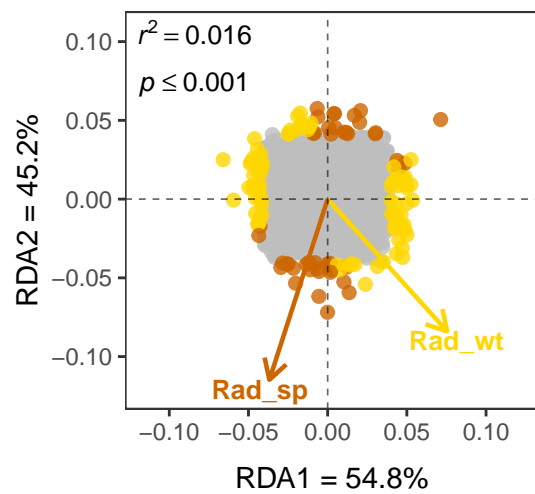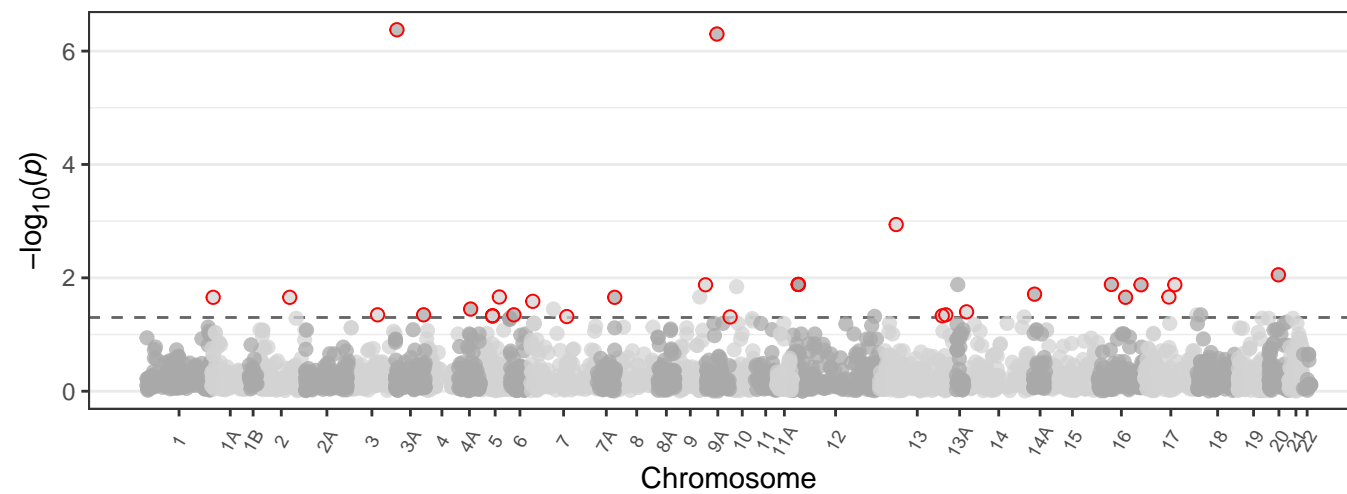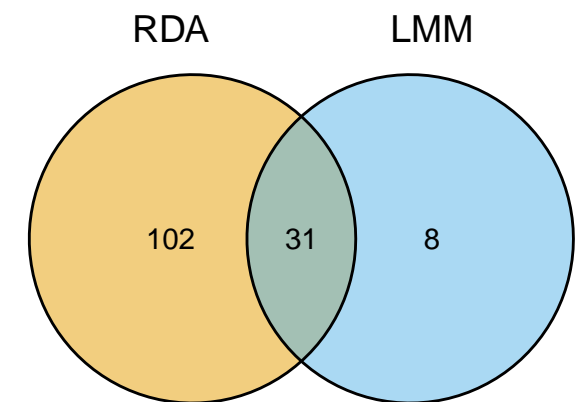

Supplement: Supplementary file 5 — Supplementary Figure S5. [file 41598_2024_59157_MOESM5_ESM.pdf]

# SN – 9587 CNVs

Temperature

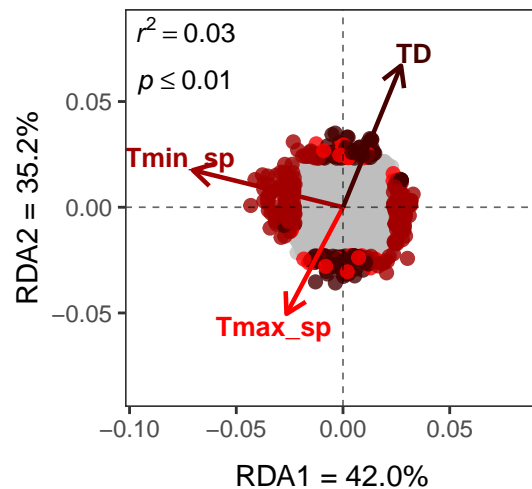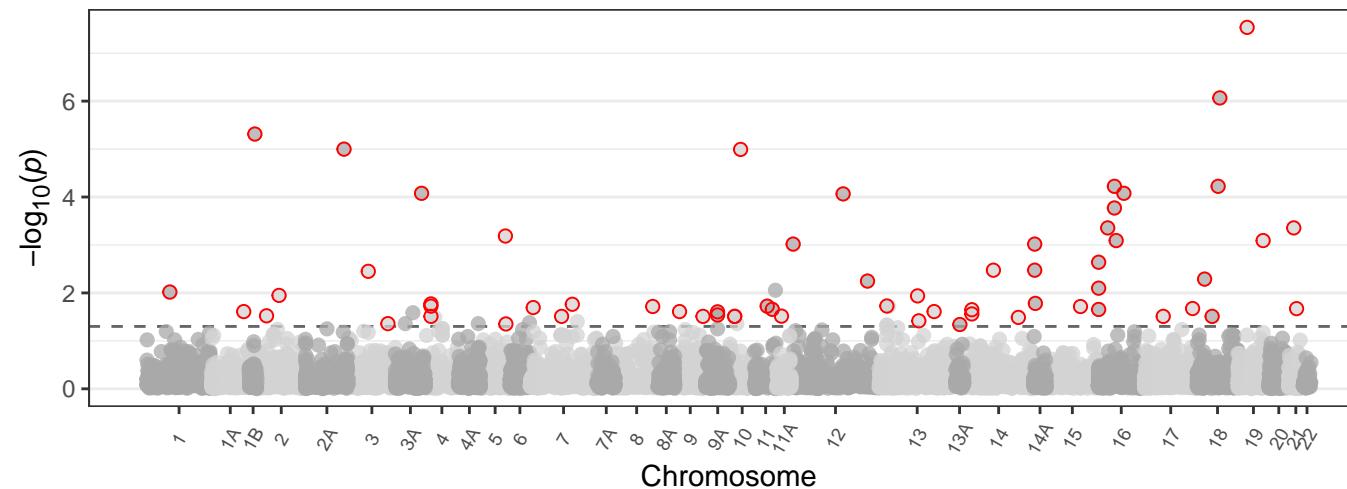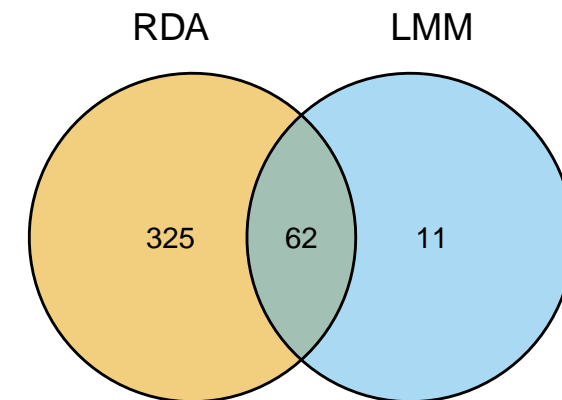

Precipitation

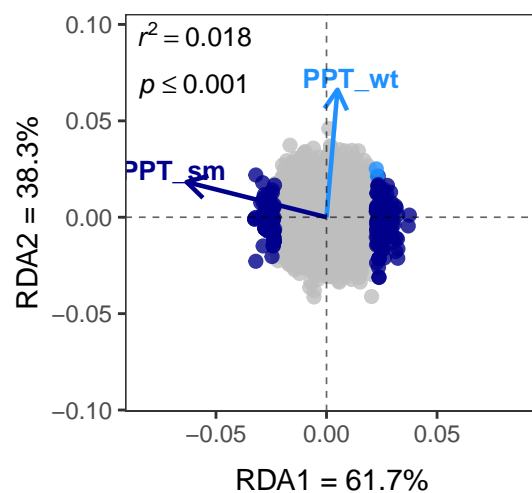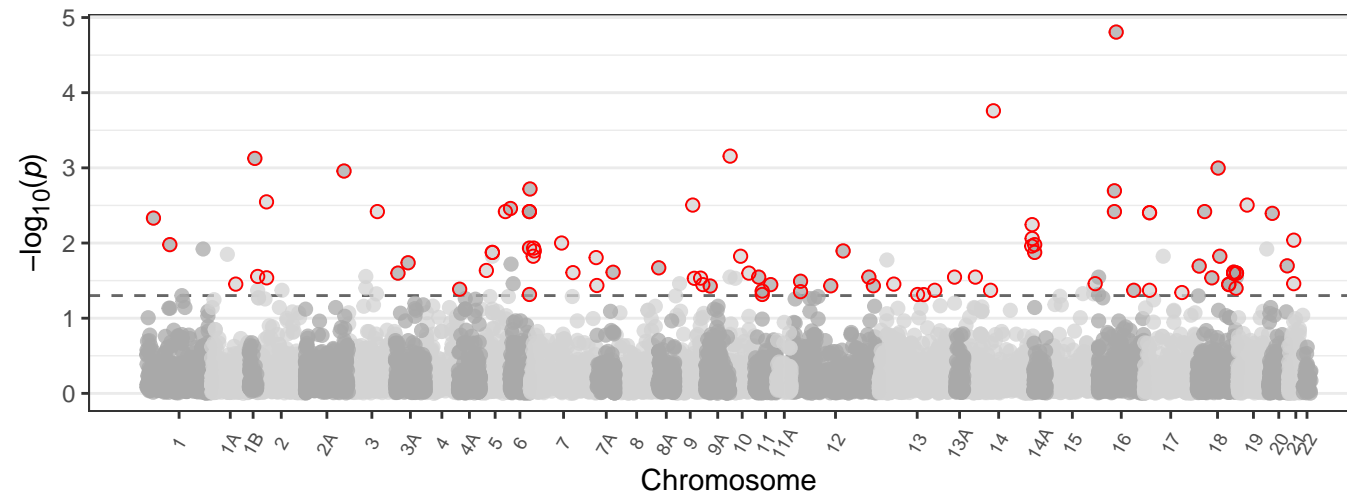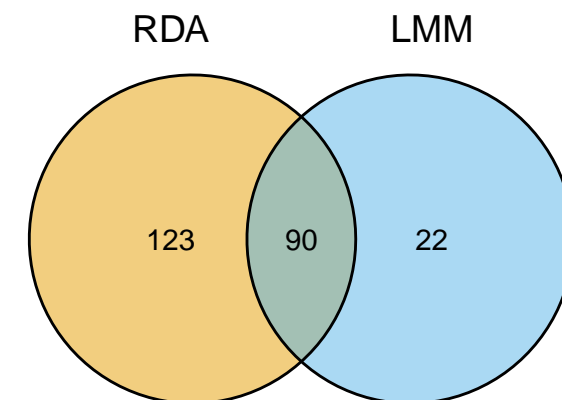

Solar radiation

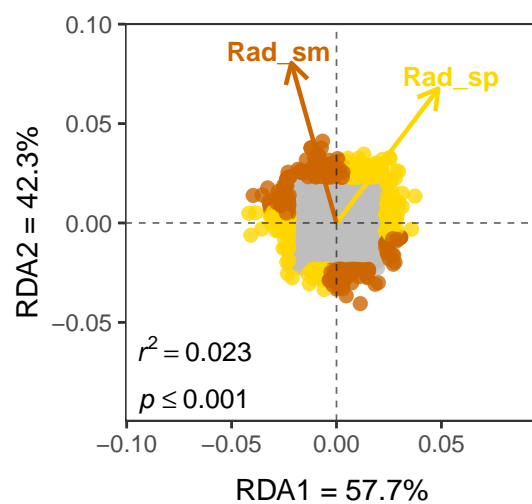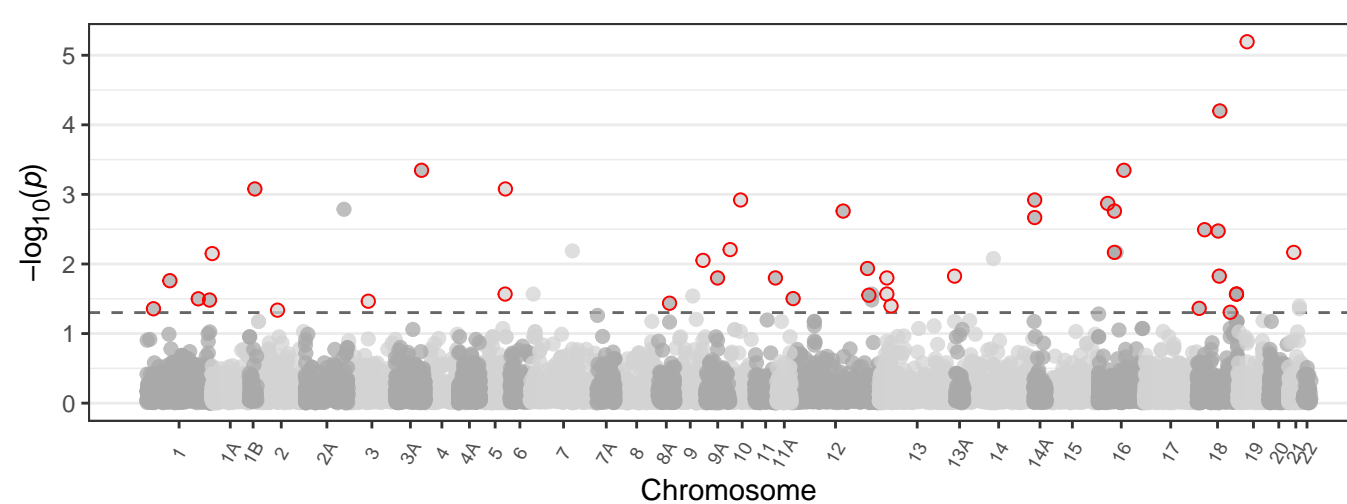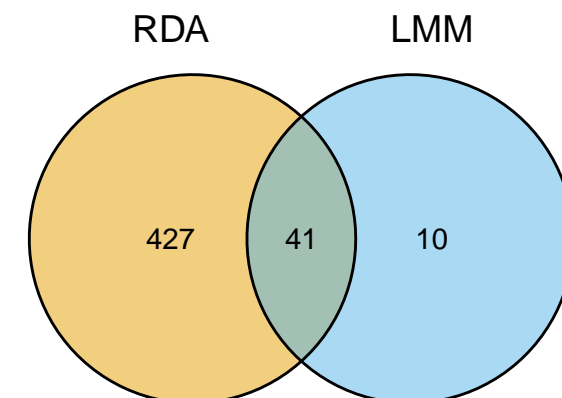

Supplement: Supplementary file 6 — Supplementary Figure S6. [file 41598_2024_59157_MOESM6_ESM.pdf]

# CU – 5060 CNVs

Temperature

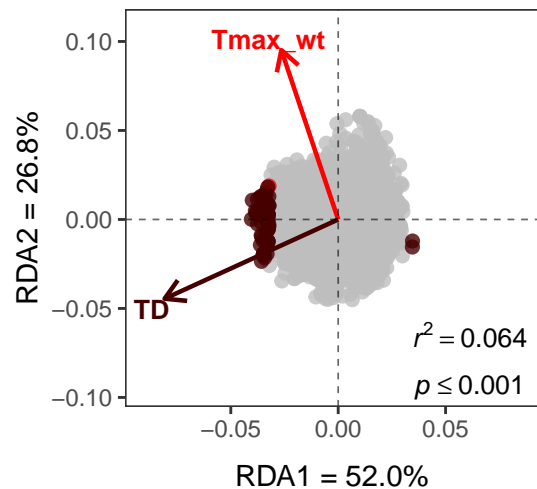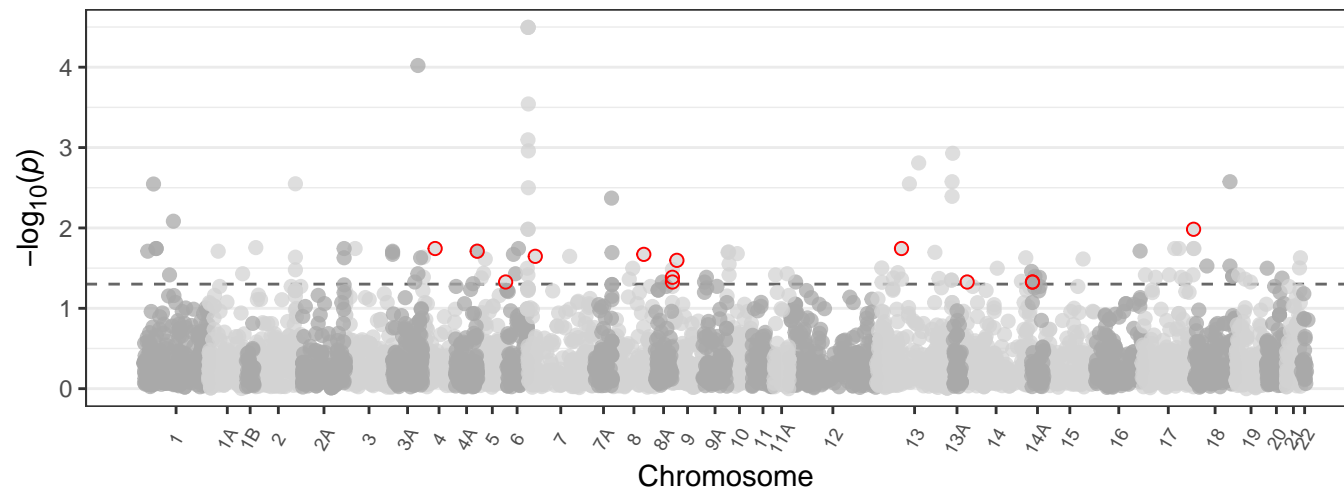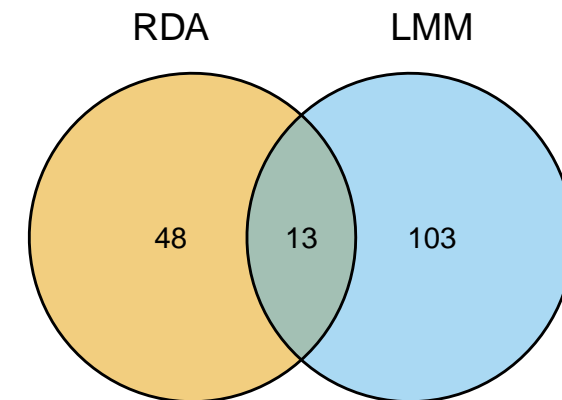

Precipitation

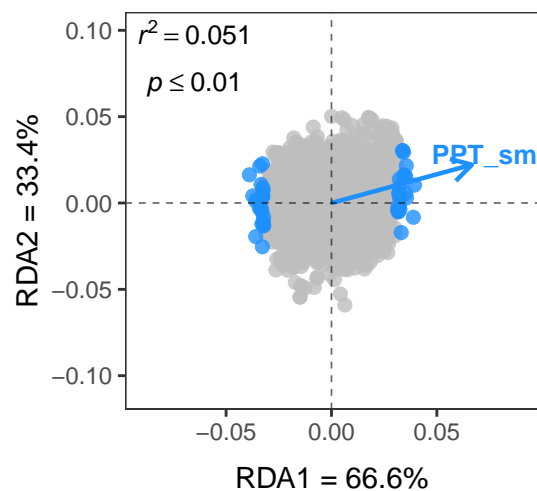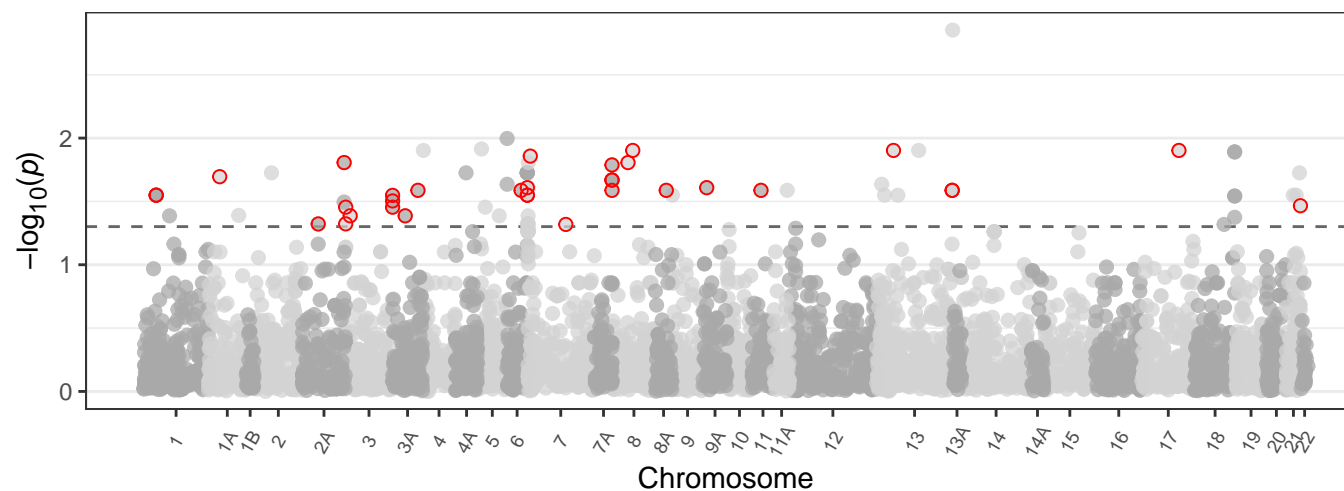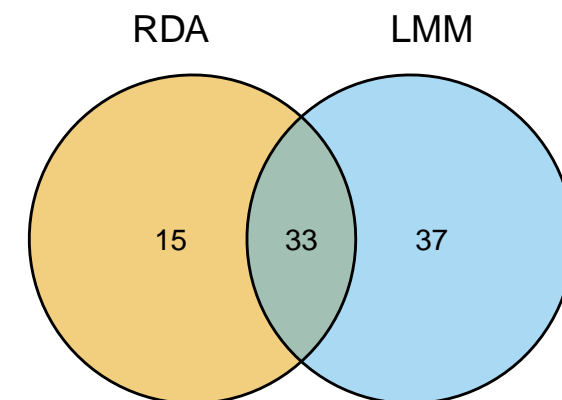

Solar radiation

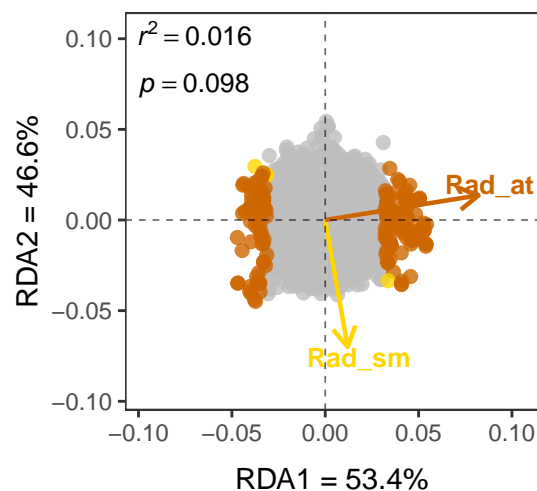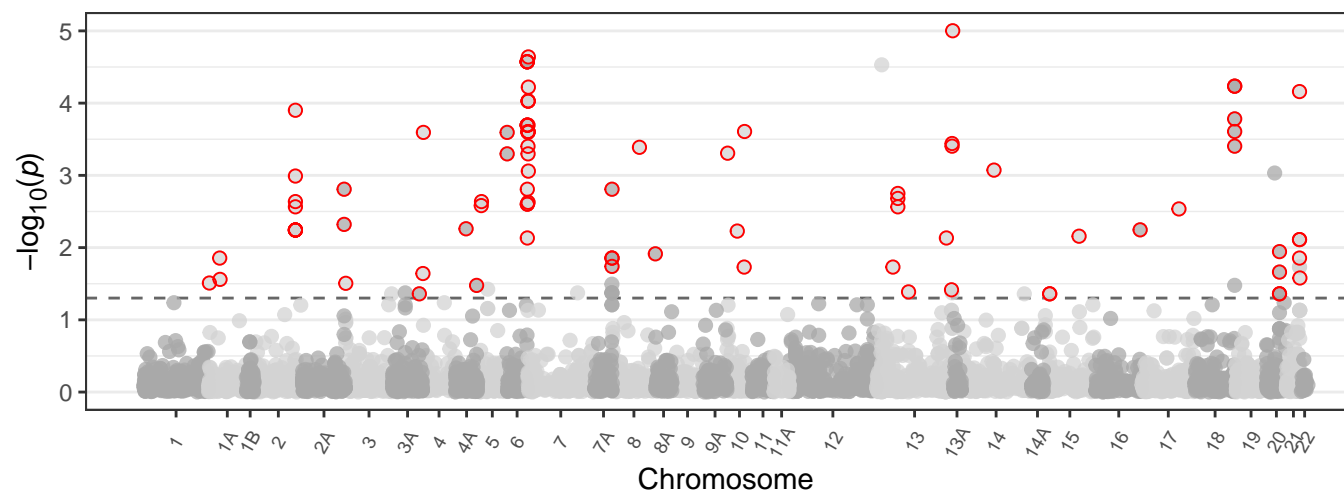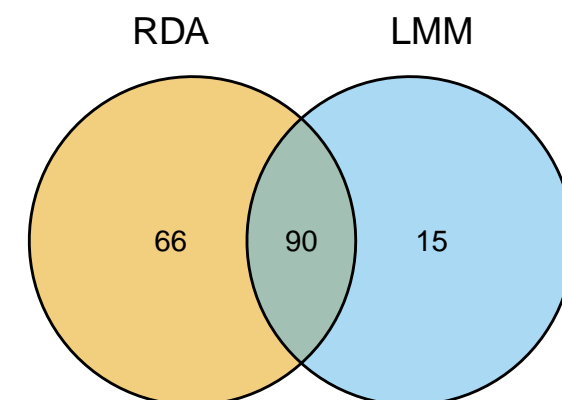

Supplement: Supplementary file 7 — Supplementary Figure S7. [file 41598_2024_59157_MOESM7_ESM.pdf]

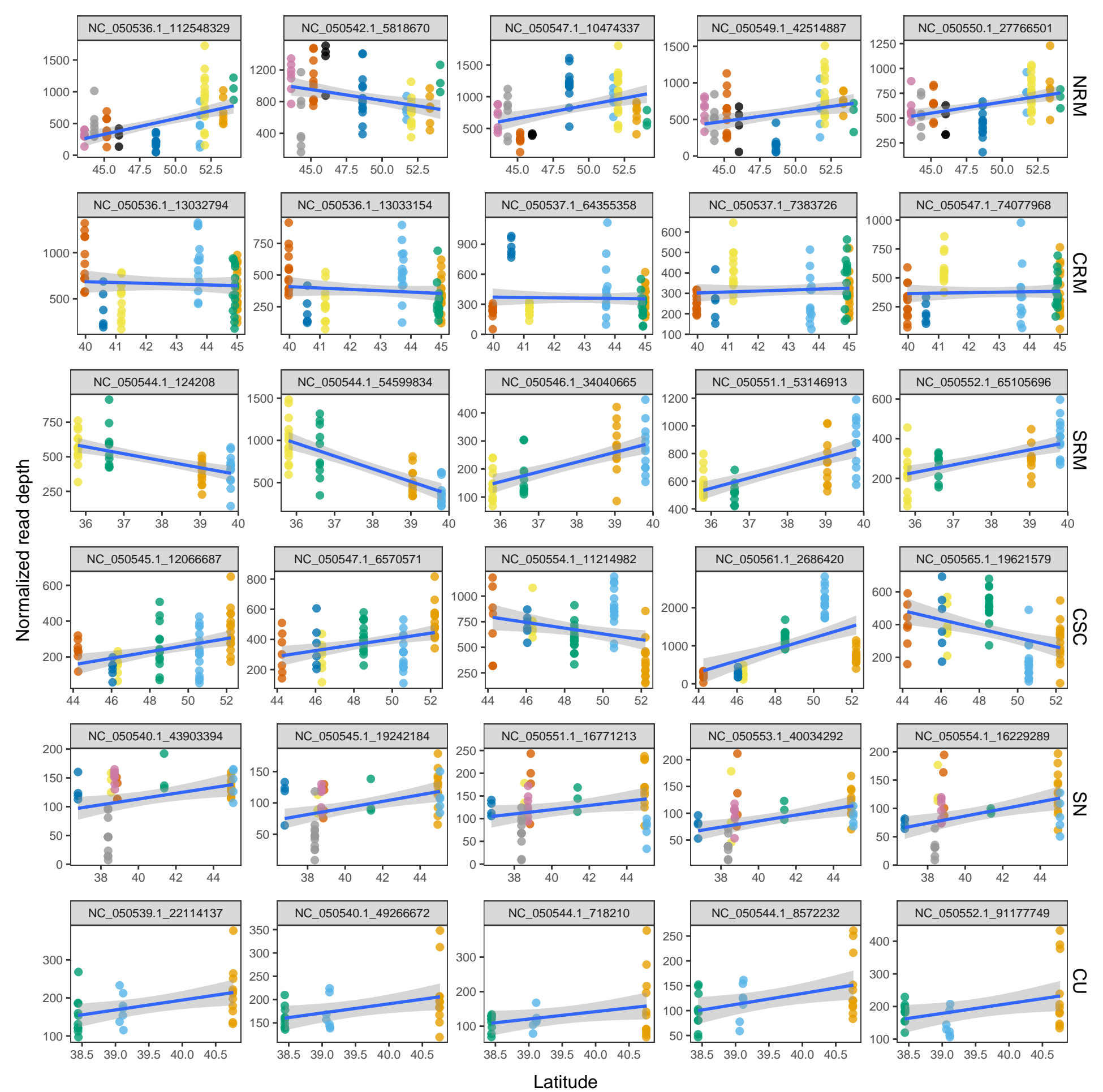

Supplement: Supplementary file 8 — Supplementary Figure S8. [file 41598_2024_59157_MOESM8_ESM.pdf]

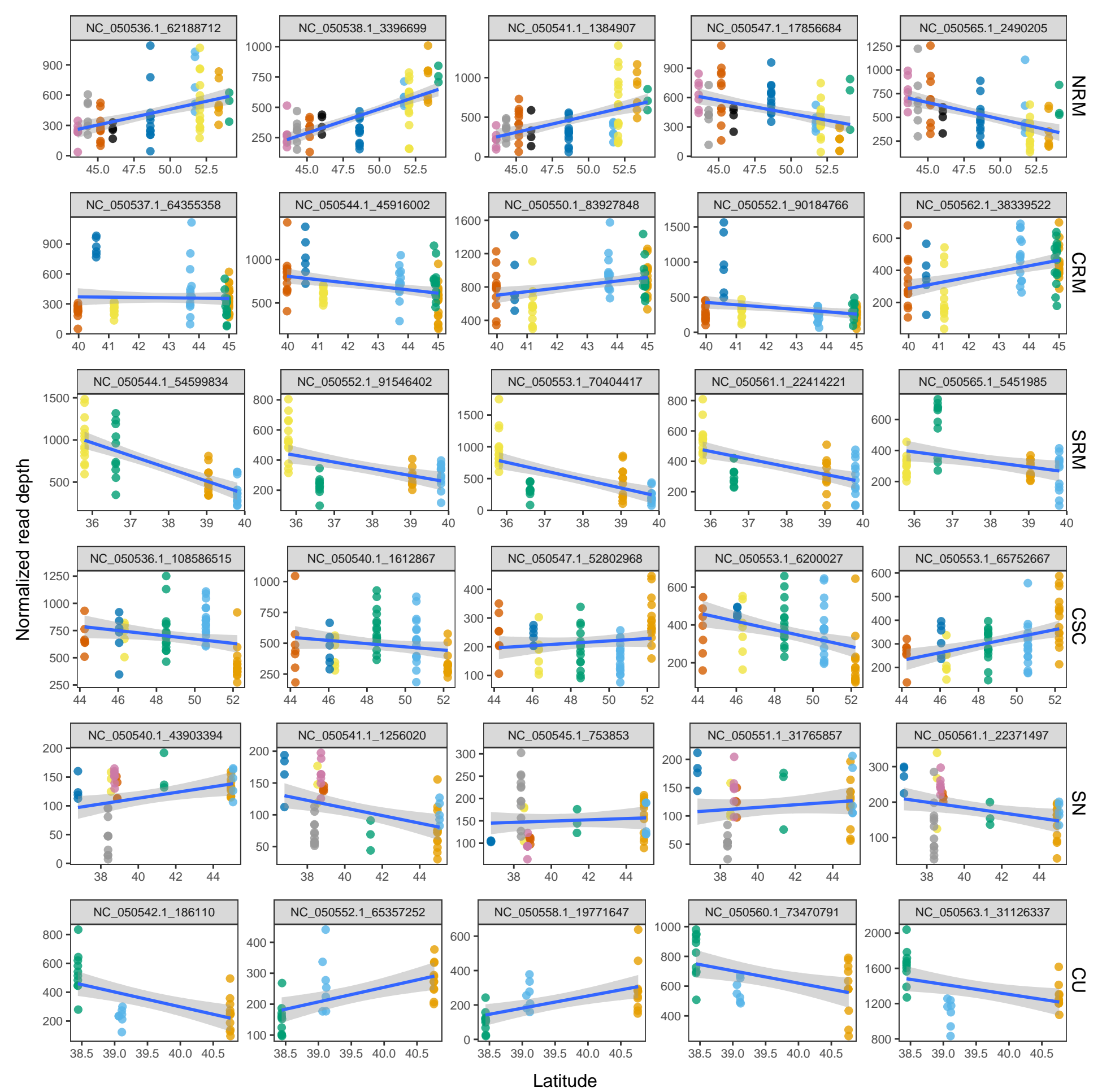

Supplement: Supplementary file 9 — Supplementary Figure S9. [file 41598_2024_59157_MOESM9_ESM.pdf]

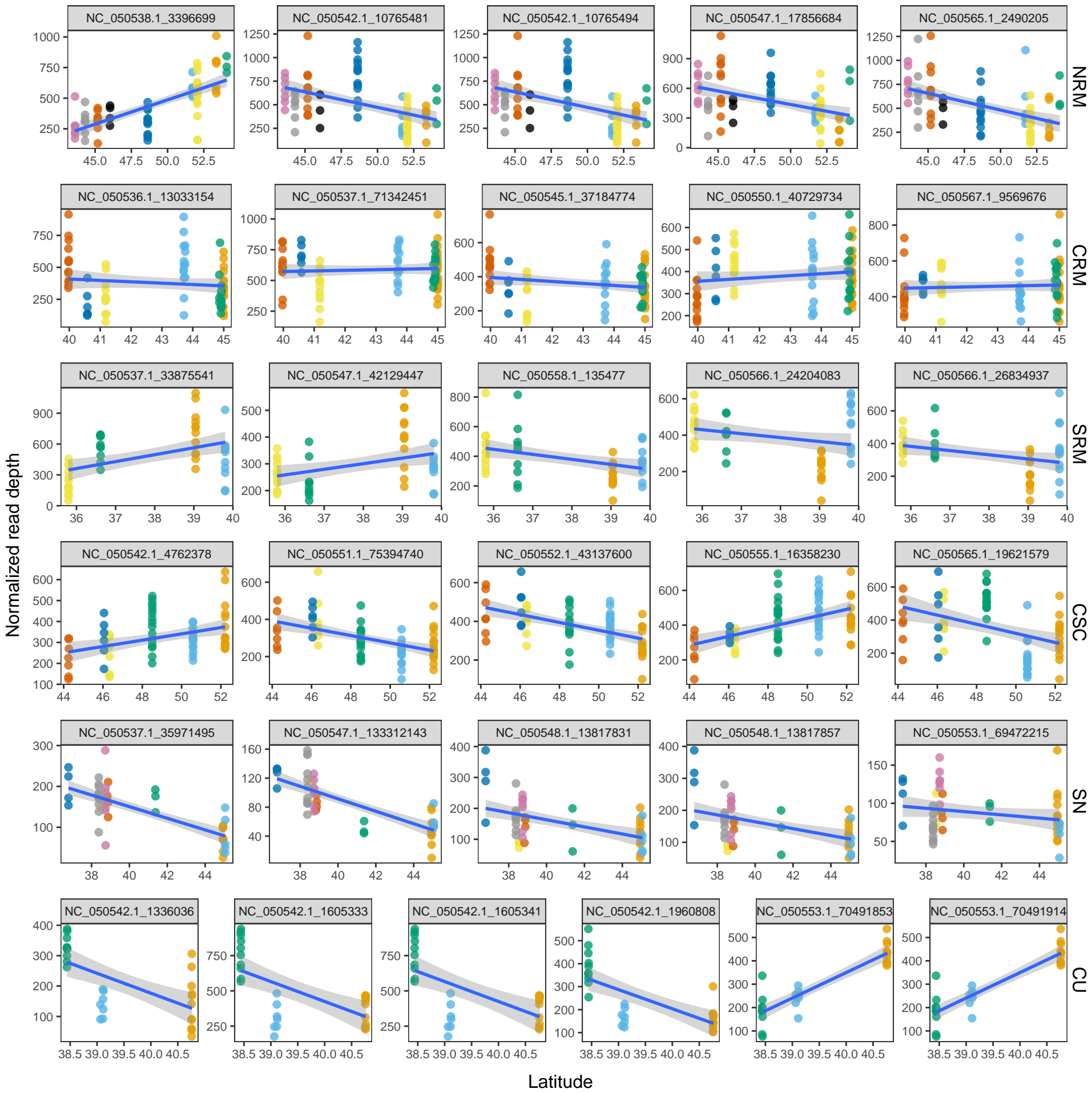

Supplement: Supplementary file 10 — Supplementary Figure S10. [file 41598_2024_59157_MOESM10_ESM.pdf]

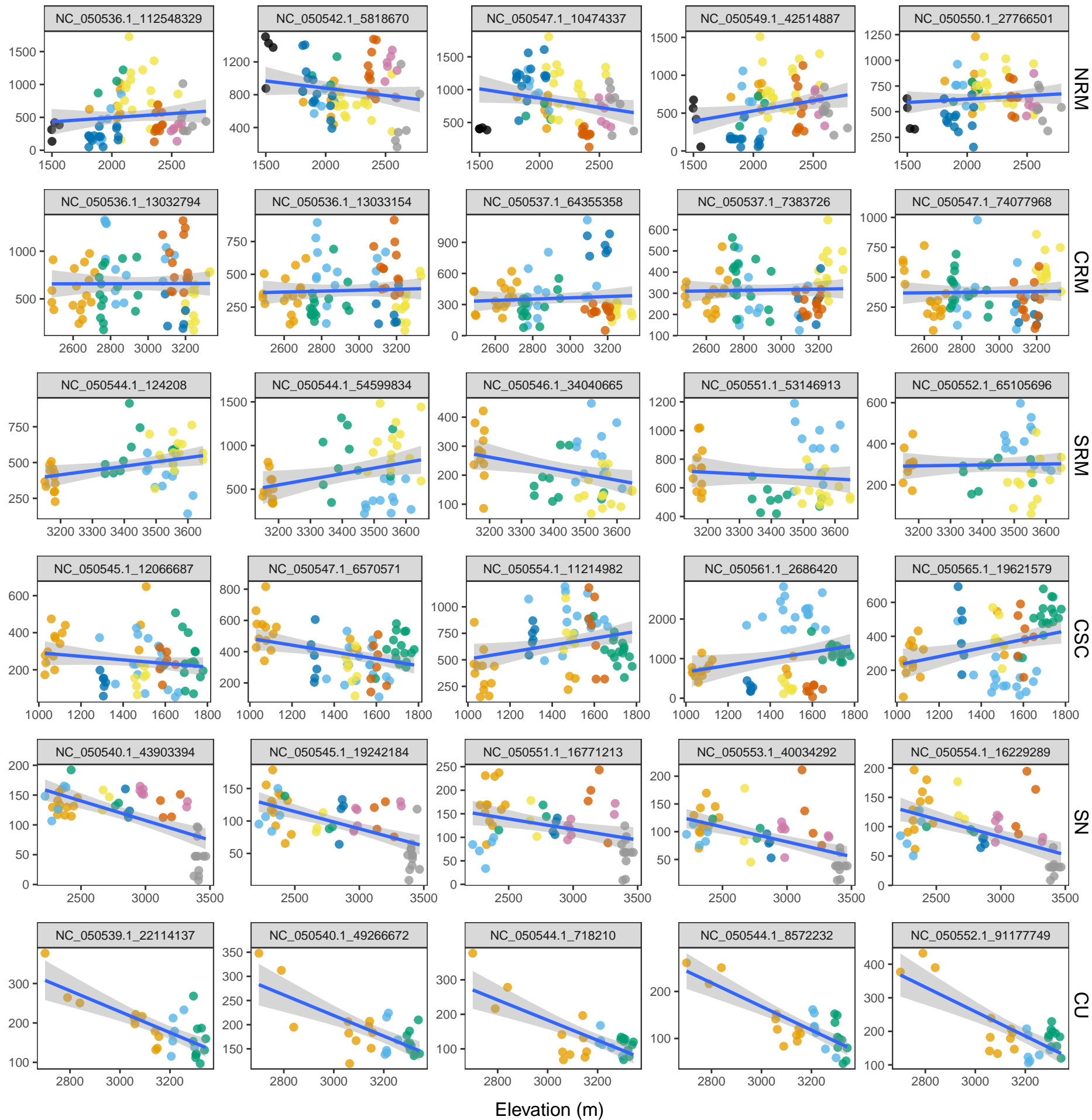

Supplement: Supplementary file 11 — Supplementary Figure S11. [file 41598_2024_59157_MOESM11_ESM.pdf]

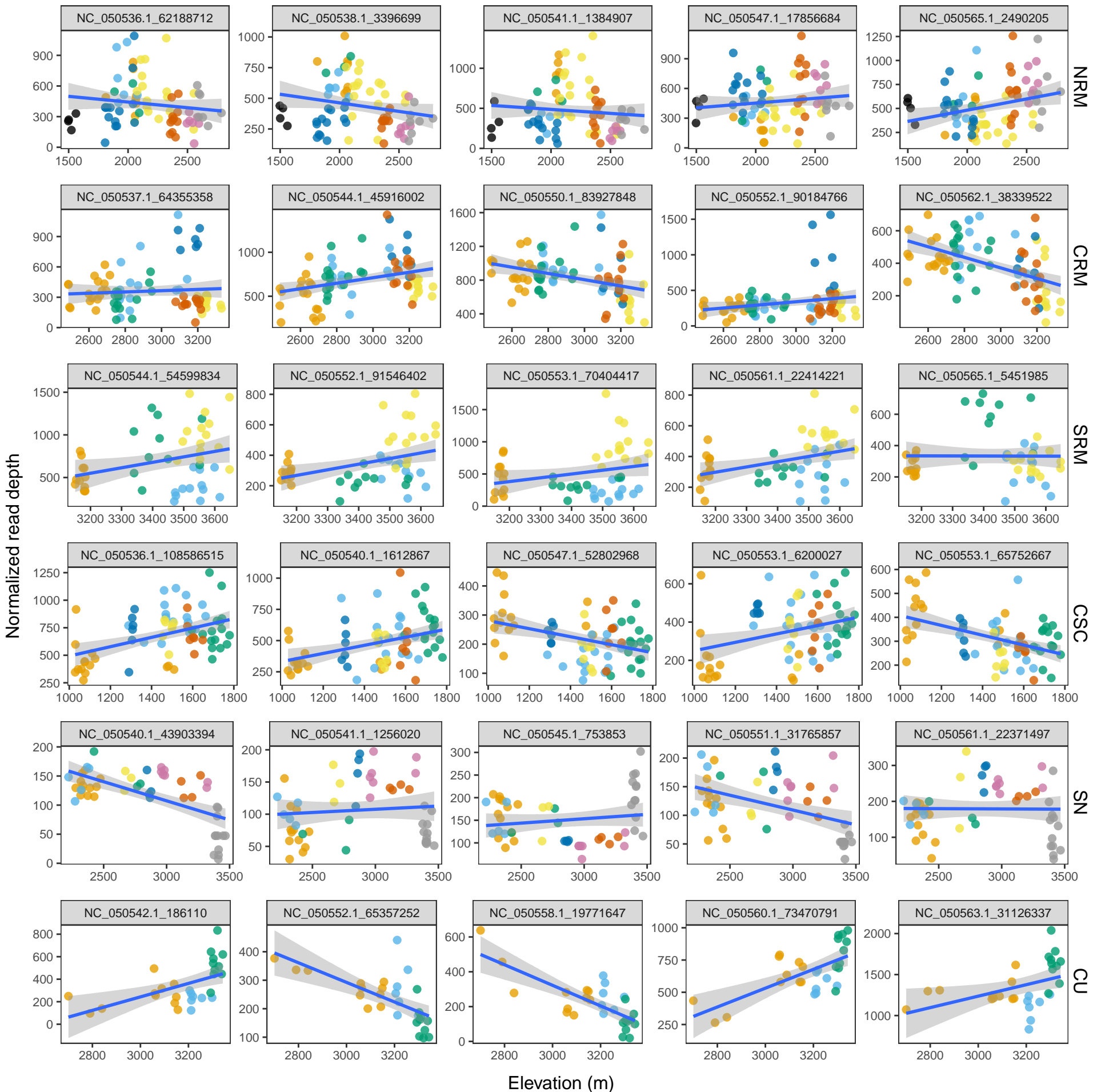

Supplement: Supplementary file 12 — Supplementary Figure S12. [file 41598_2024_59157_MOESM12_ESM.pdf]

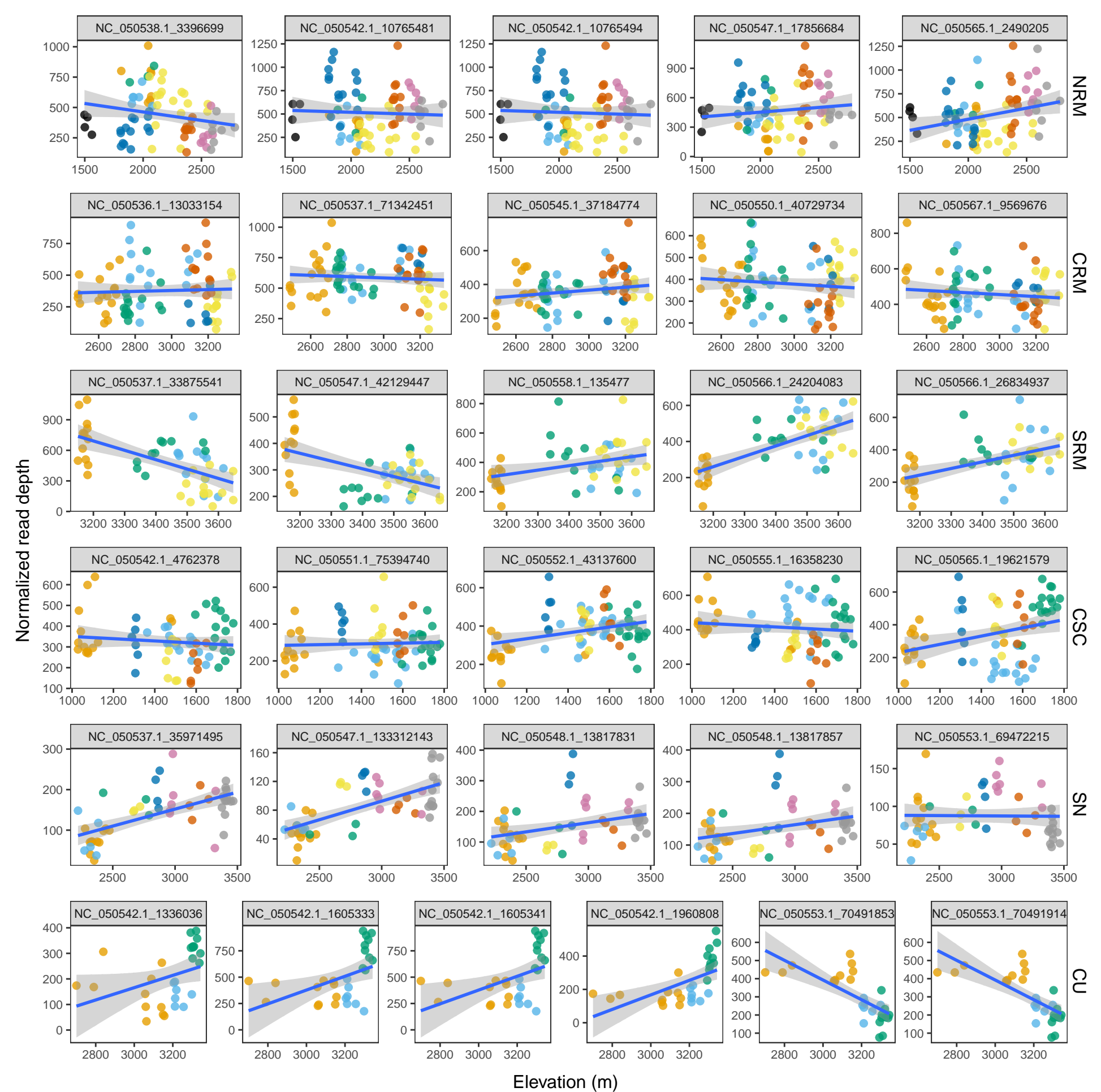

Supplement: Supplementary file 13 — Supplementary Figure S13. [file 41598_2024_59157_MOESM13_ESM.pdf]
