# Supplementary material for: Putative climate adaptation in American pikas (Ochotona princeps) is associated with copy number variation across environmental gradients
Source: Sci Rep. 2024 Apr 13;14:8568. doi: 10.1038/s41598-024-59157-6 (PMC11014952; doi:10.1038/s41598-024-59157-6)
Supplement: Supplementary file 14 — Supplementary Legends. [file 41598_2024_59157_MOESM14_ESM.docx]

**Supplementary Figure Legends**

Figure S1. PCA showing sequencing batch effect within each lineage. For all plots, colours indicate different sequencing libraries and shapes indicate sampling locations. Abbreviations are as follows: (NRM) Northern Rocky Mountains; (CRM) Central Rocky Mountains; (SRM) Southern Rocky Mountains; (CSC) Cascades; (SN) Sierra Nevada; and (CU) Central Utah.

Figures S2-S7. Outlier detection for redundancy analysis (RDA; left) and linear mixed models (LMM; middle) for six American pika lineages. For the RDA plots, outliers are coloured by which the strongest correlated variable. Hollow red circles on the Manhattan plots (middle) indicate robust outliers; only the lowest *p*-value for each locus is shown. Venn diagrams (right) show the number of detected outliers within and across methods.

Figures S8-S10. Relationship between latitude (decimal degrees) and normalized read depth of outlier loci significantly associated with temperature (Figure S8), precipitation (Figure S9), or solar radiation (Figure S10) detected during GEA analyses in American pika. Each line indicates a separate lineage, and the top five most strongly correlated loci within each lineage are shown. Abbreviations are as follows: (NRM) Northern Rocky Mountains; (CRM) Central Rocky Mountains; (SRM) Southern Rocky Mountains; (CSC) Cascades; (SN) Sierra Nevada; and (CU) Central Utah.

Figure S11-S13. Relationship between elevation and normalized read depth of outlier loci significantly associated with temperature (Figure S11), precipitation (Figure S12), or solar radiation (Figure S13) detected during GEA analyses in the American pika (*Ochotona* *princeps*). Each line indicates a separate lineage, and the top five most strongly correlated loci within each lineage are shown. Abbreviations are as follows: (NRM) Northern Rocky Mountains; (CRM) Central Rocky Mountains; (SRM) Southern Rocky Mountains; (CSC) Cascades; (SN) Sierra Nevada; and (CU) Central Utah.
